# Ionizing Radiation Actively Reshapes Bone Marrow-Derived Extracellular Vesicle MicroRNA Cargo with the Involvement of hnRNP A2b1

**DOI:** 10.3390/ijms27125510

**Published:** 2026-06-18

**Authors:** Ilona Barbara Csordás, Martina Forgács, Tünde Szatmári, Katalin Balázs, Éva Moussong, Tamás Visnovitz, Christophe Badie, Katalin Lumniczky

**Affiliations:** 1 Department of Radiobiology and Radiohygiene, National Centre for Public Health and Pharmacy, Albert Flórián út 2–6, 1097 Budapest, Hungary; csordas.ilona@phd.semmelweis.hu (I.B.C.); forgacs.martina@nngyk.gov.hu (M.F.); szatmari.tunde@nngyk.gov.hu (T.S.); balazskatus2@gmail.com (K.B.); moussong.eva@nngyk.gov.hu (É.M.); 2 Doctoral College of Pathological Sciences, Semmelweis University, Üllői út 26, 1085 Budapest, Hungary; 3 Department of Genetics, Cell and Immunobiology, Semmelweis University, Üllői út 26, 1085 Budapest, Hungary; visnovitz.tamas@semmelweis.hu; 4 Department of Plant Physiology and Molecular Plant Biology, ELTE Eötvös Loránd University, Pázmány Péter Sétány 1/c, 1117 Budapest, Hungary; 5 Centre for Radiation, Chemical and Environmental Hazards, UK Health Security Agency, Chilton, Didcot OX11 0RQ, UK; christophe.badie@ukhsa.gov.uk

**Keywords:** ionizing radiation, extracellular vesicles, exosomes, microRNAs, miRNAs, RNA-binding proteins, hnRNP A2b1, intercellular signaling, bystander effect

## Abstract

Bone marrow (BM) is highly sensitive to ionizing radiation: high doses cause extensive cell death, BM failure, and immune suppression, whereas low doses may increase long-term cancer risk without acute toxicity. Radiation-induced BM effects are partly mediated by disrupted intercellular communication via extracellular vesicles (EVs), including alterations in their microRNA cargo. EV–microRNA packaging remains unclear, although RNA-binding proteins are thought to contribute. To address this, murine BM cells and EVs were isolated 24 h after total body irradiation (0, 0.1, or 3 Gy). MicroRNAs were analyzed using nCounter and validated by RT–qPCR, while RNA-binding proteins (hnRNP A2b1, hnRNP Q) were assessed by Western blotting and confocal microscopy. Protein–microRNA interactions were examined using motif analysis and immunoprecipitation, and functional associations were explored via KEGG pathway analysis. High-dose irradiation induced widespread microRNA changes, whereas low-dose irradiation had minimal effects. Distinct cellular and EV microRNA profiles indicated selective sorting, with specific microRNAs enriched in cells but depleted in EVs. hnRNP A2b1 emerged as a potential regulator, showing nuclear relocalization and reduced EV association after irradiation; these changes correlated with decreased export of motif-containing microRNAs, possibly linked to key BM pathways. Overall, radiation alters EV–microRNAs through dose-dependent, protein-mediated selective sorting, potentially affecting BM communication and homeostasis.

## 1. Introduction

Ionizing radiation (IR) is a well-known carcinogen that can induce a variety of cellular alterations involved in carcinogenesis, including DNA damage, oxidative stress, and inflammation. BM exposure to IR can lead to the development of hematological malignancies, such as acute myeloid leukemia (AML) [1]. To mitigate the long-term consequences of IR exposure and to develop preventive strategies, it is crucial to understand the underlying molecular mechanisms involved in these processes.

In the BM, hematopoietic cells develop from hematopoietic stem cells (HSCs) in a specialized and supportive microenvironment (ME). Cells within the BM ME are continuously interconnected with each other and with HSCs, providing optimal conditions for healthy hematopoiesis. IR exposure disrupts BM function by directly damaging BMCs and interfering with intercellular communication within the BM niche, leading to both short- and long-term consequences. In addition to direct damage, IR also induces changes in nonirradiated cells, a phenomenon known as the radiation-induced bystander effect (RIBE). This effect refers to radiation-induced alterations in cells not directly exposed to radiation. While the precise mechanisms underlying RIBE remain unclear, intercellular communication plays a crucial role in this process [2,3]. In this context, biological structures involved in intercellular communication, such as cytokines, chemokines, small molecules, and extracellular vesicles, should be investigated. In the present study, we focused on EVs.

EVs are nano- to micro-sized particles, released by cells into the extracellular environment and are characterized by their lipid membrane bilayer structure and absence of a functional nucleus, making them unable to replicate [4]. These vesicles participate in intercellular communication by delivering complex bioactive cargo, such as lipids, proteins, DNA, and coding and noncoding RNA, such as microRNAs (miRNAs), to EV recipient cells [5]. The cargo composition and secretion rate of EVs depend on the status of the donor cells. Cellular stress, such as IR exposure, can enhance EV release and alter the composition of EV cargo [6,7,8]. One of the key components of EV cargo affected by IR exposure is their miRNA pool. These short noncoding RNAs play a key role in posttranscriptional regulation of cellular pathways and processes. We previously reported that miRNAs are involved in the BM IR response and the development of RIBE [9].

Although the importance of the miRNA cargo of EVs is recognized, the exact mechanism by which miRNAs are incorporated into EVs remains unclear. The sorting and encapsulation processes are of great interest for understanding EV functions, identifying biomarkers, and enhancing RNA therapeutic delivery [10]. Initially, the sorting of miRNAs into EVs was thought to be a random process. However, an increasing number of studies showed that the miRNA profile of EVs resulted from an active sorting mechanism rather than a passive process. Although the exact miRNA-loading machinery has not yet been mapped, the role of RNA-binding proteins (RBPs) in this process has already been demonstrated [10,11,12]. RBPs are proteins involved in posttranscriptional regulation by interacting with RNAs via specific RNA-binding domains (RBDs). RBDs recognize and bind to specific short nucleotide sequences (motifs) in miRNAs and participate in the synthesis, cellular distribution, and vesicular transport of miRNAs [13,14].

Previously, we developed an in vivo mouse model to study the role of EVs in BM function and radiation response, including the development of radiation-induced bystander effects [8,15]. In this model, EVs isolated from the BM of directly irradiated mice were injected into naïve (nonirradiated) mice, in which we could follow in vivo radiation-induced bystander effects transmitted by EVs. Using this approach, we observed an overall increase in BM EV secretion and changes in the EV phenotype [8,16]. EVs originating from irradiated mice were capable of inducing bystander effects in the BM, spleen, and blood of recipient animals [8,15,17]. We further demonstrated that BM-derived EVs not only altered hematopoietic and immune cell subsets in ways resembling direct irradiation but also induced DNA damage and cell death [8,15], enhanced oxidative stress [17], and changed plasma protein profiles associated with inflammation and immune responses [18]. It was demonstrated that only EVs isolated shortly after IR could induce bystander responses; nevertheless, EV-transmitted effects were persistent [8]. BM cells (BMCs) treated with EVs from irradiated BM exhibited proteomic changes mirroring the proteomic alterations found in directly irradiated cells. These proteomic alterations were partially linked to the regulatory function of miRNAs delivered by the EVs, underscoring their role in mediating RIBE [9]. In certain cell populations (e.g., hematopoietic stem and progenitor cells, HSPCs), regeneration was slower in EV-injected mice than in directly irradiated ones [8].

The current study aimed to investigate how IR influenced BM-derived EV–miRNA cargo, with a focus on the role of RBPs.

## 2. Results

### 2.1. Ionizing Radiation Alters miRNA Composition in Bone Marrow-Derived Extracellular Vesicles, Potentially Through Regulating RNA-Binding Proteins

**Figure 1:**
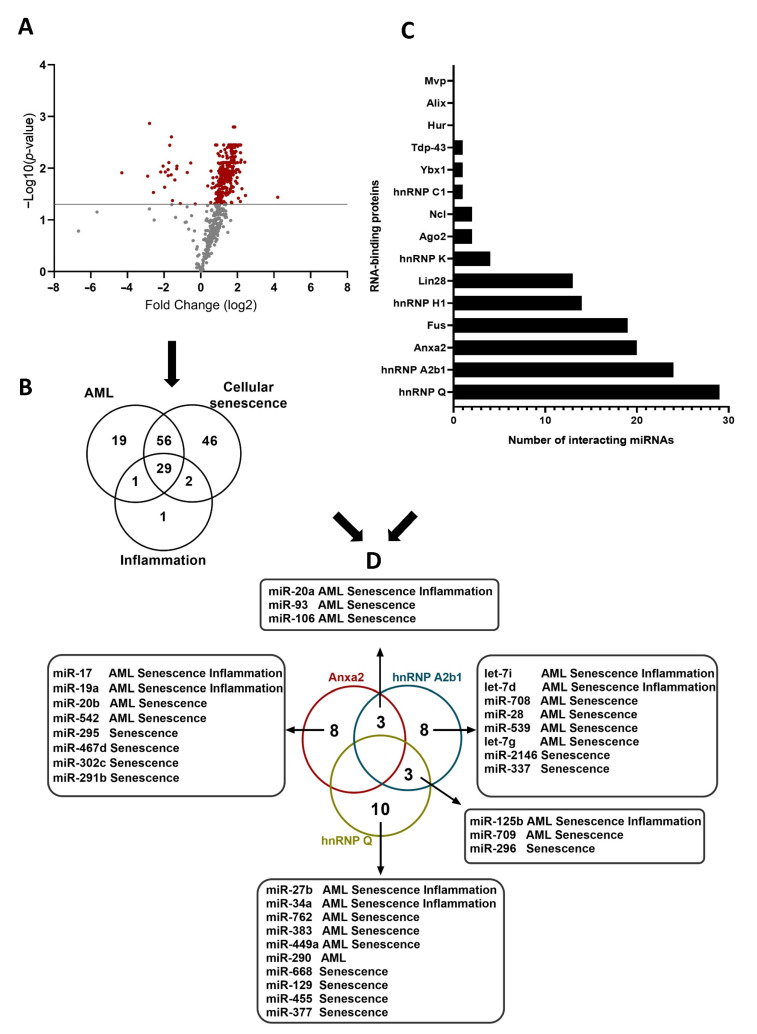
3 Gy deregulated EV–miRNAs bind RNA-binding proteins and affect leukemia, senescence, and inflammation pathways. (**A**) A volcano plot displaying differentially expressed miRNAs in BM-EVs 24 h after 3 Gy irradiation, compared to control (0 Gy) BM-EV miRNAs. The volcano plot is based on log2-fold change and −log10 *p*-values derived from nCounter data (as detailed in Materials and Methods). Statistically significant miRNAs (adjusted *p*-value < 0.05, Benjamini–Hochberg method) are highlighted in red; the gray line represents the FC threshold. (**B**) Venn diagram identifying significantly altered miRNAs (adjusted *p*-value < 0.05) associated with acute myeloid leukemia, cellular senescence, or inflammation following 3 Gy irradiation. Functional annotation was performed using KEGG and GO databases, as well as relevant literature. (**C**) Bar graph displaying the number of interactions between miRNAs and RNA-binding proteins. These interactions were identified through miRNA sequence analysis, where specific binding motifs were examined. Only miRNAs with a log2-fold change < −1.5 or >1.5 (adjusted *p*-value < 0.05) were considered. The number of miRNAs interacting with each protein is shown. (**D**) Distribution chart illustrating how miRNAs containing binding motifs for hnRNP A2b1, hnRNP Q, or Anxa2 are associated with acute myeloid leukemia, senescence, and inflammation pathways.

The miRNA profile of BM-derived EVs was studied by NanoString analysis, which revealed that EVs from mice irradiated with 3 Gy had 326 significantly deregulated miRNAs 24 h after IR compared to EVs from sham-irradiated mice (Figure 1A).

A database analysis was performed to identify, among all 326 deregulated miRNAs, those associated with AML or with processes related to AML development, such as cellular senescence or inflammation, since the main long-term consequence of BM irradiation is the development of hematological malignancies. The analysis revealed that 154 miRNAs were associated with one or more of these pathways, of which 105 miRNAs, 133 miRNAs, and 33 miRNAs were related to AML, cellular senescence, and inflammation, respectively (Figure 1B).

It has been reported that miRNA packaging into EVs is carried out by multiple RNA-binding proteins, which recognize specific nucleotide motifs (recognition motifs) within the miRNA sequence (studies listed in Supplementary Table S1). Fifteen RBPs with reported roles in sorting and packaging miRNAs into EVs were selected for further analysis (Supplementary Table S1). To predict miRNA–RBP interactions, RBP recognition motifs were scanned within the sequences of the 326 deregulated miRNAs, resulting in the identification of 130 potential interactions. These interactions involved 87 different miRNAs and 12 RBPs. Among the interacting miRNAs, 71% (62 out of 87) were associated with three key proteins: heterogeneous nuclear ribonucleoprotein Q (hnRNP Q/SYNCRIP/NSAP–1), which interacts with 29 miRNAs; heterogeneous nuclear ribonucleoprotein A2b1 (hnRNP A2b1), which interacts with 24 miRNAs; and Annexin A2 (Anxa2), which interacts with 20 miRNAs (Figure 1C). Thirty-two of these miRNAs were identified to play a role in processes associated with AML, cellular senescence, or inflammation (Figure 1D). Most of these miRNAs interacted exclusively with one of the three proteins, but a subset could bind to two proteins (hnRNP A2b1 with Anxa2 or hnRNP A2b1 with hnRNP Q), suggesting a moderate level of redundancy in miRNA binding by RBPs.

### 2.2. Ionizing Radiation Modulates RNA-Binding Protein Levels in Bone Marrow and Their Extracellular Vesicles and Promotes hnRNP A2b1 Nuclear Relocalization

**Figure 2:**
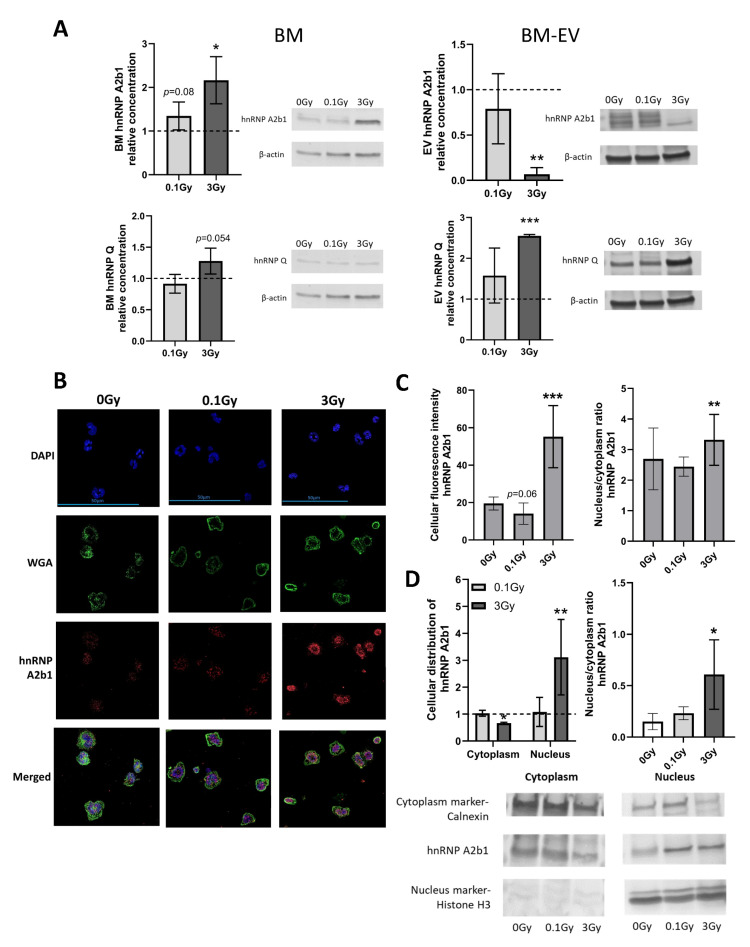
Ionizing radiation alters bone marrow RNA-binding proteins concentrations, cellular localization, and EV packaging (**A**) Western blot analysis of the hnRNP A2b1 and hnRNP Q proteins in the BM and BM-derived extracellular vesicles 24 h after irradiation with 0.1 Gy or 3 Gy. Forty micrograms of protein were loaded on an SDS-polyacrylamide gel, marked with anti-hnRNP A2b1 or anti-hnRNP Q antibodies, and visualized with secondary antibody binding and DAB staining. β-actin was used as a loading control. Quantification was performed with ImageJ software version 1.54g (***n*** = 3). Data are normalized to the respective control samples; the dashed line indicates the control levels set to 1. Replicates are presented in Supplementary Figures S4 and S5. (**B**) Confocal microscopic analysis of hnRNP A2b1 in control, 0.1 Gy- and 3 Gy-irradiated BM cells (***n*** = 100 cells/experiment). BM cells were stained with DAPI (blue, nucleus), WGA (green, cell membrane), and hnRNP A2b1 antibodies (red). (**C**) Cellular fluorescence intensity of hnRNP A2b1 (**left**) and the nucleus/cytoplasm ratio of hnRNP A2b1 (**right**) calculated from confocal microscopy images by ImageJ. (**D**) Western blot validation of the cellular redistribution of hnRNP A2b1 24 h after IR exposure in the BM. Nuclear- and cytoplasm-enriched fractions were extracted, and the changes in the hnRNP A2b1 protein concentration were assessed by Western blot analysis. Calnexin was used as a cytoplasmic marker, and histone H3 was used as a nuclear marker. The cellular distribution (**left**, normalized to the respective control samples) and nucleus/cytoplasm ratios (**right**) were calculated from the blots with ImageJ (***n*** = 3, replicates are presented in Supplementary Figure S6). For statistical analysis, Student’s *t*-test was used, and *p* < 0.05 was considered significant. Significance levels are denoted by asterisks: * for *p* < 0.05, ** for *p* ≤ 0.01, *** for *p* ≤ 0.001.

Since hnRNP A2b1, hnRNP Q, and Anxa2 had the greatest number of potential binding partners among the significantly deregulated EV-miRNAs after irradiation with 3 Gy, we investigated how IR influenced the concentration of these proteins in the EV-releasing BMCs and in the BM-derived EVs (Figure 2A and Supplementary Figure S2).

The concentration of the hnRNP A2b1 protein increased 2.1-fold in the BM (*p* = 0.012), whereas it decreased by 16.6-fold in the EVs (*p* = 0.002) (Figure 2A). The level of hnRNP Q increased 1.3-fold in BM (*p* = 0.054) and was significantly elevated in EVs by 2.6-fold (*p* = 0.0003) (Figure 2A). There were no significant alterations in the concentration of Anxa2 upon irradiation in BM or in the EVs (Supplementary Figure S2). Low-dose irradiation (0.1 Gy) did not induce significant changes in the concentrations of the three proteins in either the EVs or the BM (Figure 2A).

Overall, analysis of protein concentrations in both BM and EVs suggests the presence of an active EV protein sorting mechanism in response to high-dose (3 Gy) irradiation.

To gain insight into the mechanisms regulating protein sorting into EVs, the cellular localization of hnRNP A2b1 and hnRNP Q was studied by confocal microscopy. Microscopic examination of BMCs confirmed that the level of hnRNP A2b1 was elevated by 2.81-fold (*p* = 0.00001) in the BM following exposure to 3 Gy (Figure 2B,C), correlating with Western blot analysis. Additionally, 3 Gy IR induced the intracellular relocalization of hnRNP A2b1 into the nucleus with a 1.3-fold increase in the nucleus/cytoplasm ratio (*p* = 0.007) compared to nonirradiated cells (Figure 2C). This increased nucleus/cytoplasm ratio of the hnRNP A2b1 protein was confirmed by Western blot analysis (Figure 2D). IR did not affect the intracellular localization of the hnRNP Q protein (Supplementary Figure S3).

### 2.3. Ionizing Radiation-Induced Alterations in the miRNA Cargo of Bone Marrow-Derived Extracellular Vesicles Do Not Mirror the miRNA Profile of Irradiated Donor Bone Marrow Cells

**Figure 3:**
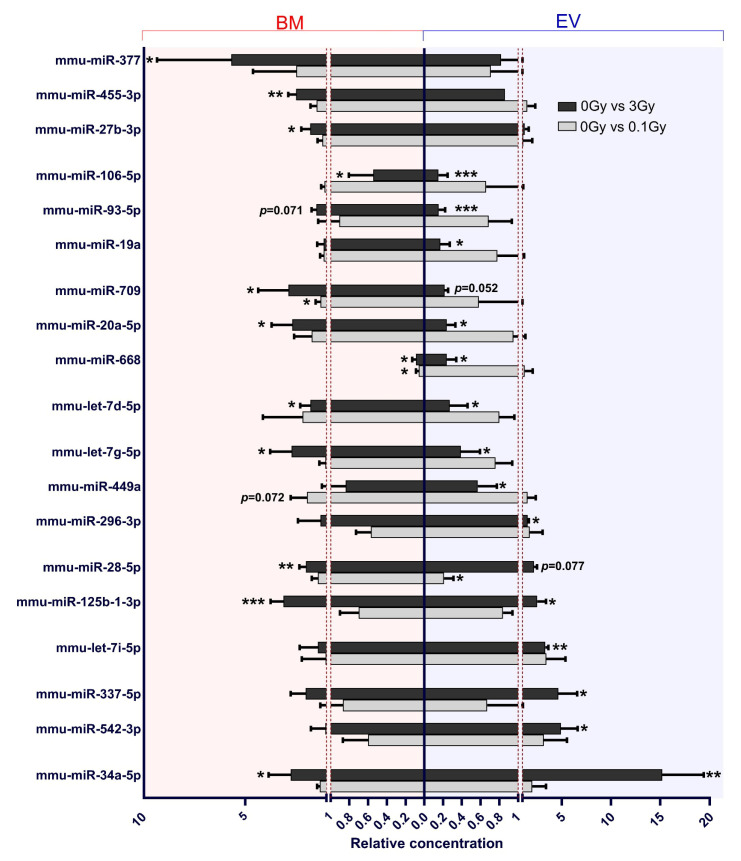
Ionizing radiation differentially alters miRNA levels in bone marrow cells and extracellular vesicles. MiRNAs were analyzed by qRT–PCR, with fold changes calculated using the ΔΔCt method as described in the materials and methods. Irradiated BMCs were compared to 0 Gy BM, and EVs from irradiated mice to EVs from control mice (***n*** = 6 for BM, ***n*** = 4 for EVs). MiRNAs with *p* < 0.05 were considered significantly deregulated, based on the Mann-Whitney U test. Data are normalized to the respective control samples, and the red dashed line indicates the control level, set to 1. Significance levels are denoted by asterisks: * for *p* < 0.05, ** for *p* ≤ 0.01, *** for *p* ≤ 0.001.

As shown in Figure 1D, 32 miRNAs differentially expressed in the EVs of BMCs irradiated with 3 Gy carried a recognition motif for the hnRNP A2b1, hnRNP Q, or Anxa2 protein and were linked to at least one of the following processes: AML, cellular senescence, or inflammation. To prioritize candidates for further study, miRNAs were chosen based on their predicted interactions with proteins showing significant changes, as well as their relevance to key cellular pathways. Since hnRNP A2b1 was greatly altered in both BM and EVs, all related miRNAs were examined. For hnRNP Q, which increased significantly in EVs, two miRNAs were analyzed from each category: senescence, senescence and AML, and those associated with all three processes (senescence, AML, and inflammation). As no significant changes were shown by Anxa2, only one miRNA per group was selected for further analysis. In total, 24 miRNAs were further analyzed by RT-qPCR, and their expression was compared between EV-donor BMCs and EVs (Figure 3).

Among the 24 assayed miRNAs, 19 were significantly altered following exposure to ionizing radiation (Figure 3). Four miRNAs (miR–129–5p, miR–708–5p, miR–383–5p, and miR–539–5p) were detectable but did not show alterations in either EVs or BM after IR (Supplementary Figure S7), and miR–302c remained undetectable.

In the BM irradiated with 3 Gy, the expression of 10 miRNAs (miR–34a–5p, miR–125b–1–3p, miR–28–5p, let–7g–5p, let–7d–5p, miR–20a–5p, miR–709, miR–27b–3p, miR–455–3p, miR–377) increased, whereas the expression of two miRNAs (miR–668 and miR–106–5p) decreased (Figure 3).

In the EVs from BM irradiated with 3 Gy, the levels of 6 miRNAs (mir–34a–5p, miR–542–3p, miR–337–5p, let–7i–5p, miR–125b–1–3p, miR–296–3p) increased, whereas those of 8 miRNAs (miR–106–5p, miR–93–5p, miR–19a, miR–20a–5p, miR–668, let–7d–5p, let–7g–5p, miR–449a) decreased (Figure 3).

Unlike high-dose exposure, low-dose irradiation had a very mild effect on the miRNA profile of both BM and BM-derived EVs. miR–709 increased and miR–668 decreased in BM, while only miR–28–5p was decreased in EVs (Figure 3).

Comparison of these profiles revealed that EV content does not merely mirror the cellular compartment. Only four miRNAs changed in the same direction in both BM and EVs (miR–106–5p and miR–668 decreased; miR–125b–1–3p and miR–34a–5p increased). Instead, we identified specific sorting patterns indicative of active cargo regulation.

A substantial group of miRNAs, broadly classified as “cell-retained” or “EV-depleted,” displayed restricted vesicular export. This group includes let–7g–5p, let–7d–5p, miR–20a–5p, and miR–709, which were upregulated in BMCs but significantly decreased or showed a downward trend (miR–709 *p* = 0.052) in EVs. Additionally, miR–19a and miR–93–5p were significantly depleted in EVs, while they were slightly increased in BMCs. Three further miRNAs (miR–27b–3p, miR–455–3p, miR–377) showed selective cellular enrichment, as their EV levels remained unchanged despite significant cellular upregulation. Collectively, these patterns suggest that despite their cellular induction or presence, these miRNAs are selectively retained within the cell rather than exported into the EV.

In contrast, the “EV-enriched” miRNAs (miR–542–3p, miR–337–5p, let–7i–5p, and miR–296–3p) remained unchanged in the cellular compartment but were significantly increased in EVs, indicating radiation-induced vesicular enrichment.

Among the 15 miRNAs that were significantly, or nearly significantly (miR–709, *p* = 0.052) altered after 3 Gy, 10 were associated with hnRNP A2b1 (miR–337–5p, let–7i, miR–125b, miR–296, let–7g, let–7d, miR–20a–5p, miR–709, miR–106–5p and miR–93–5p).

Five with hnRNP Q (miR–34a, miR–125b, miR–296, miR–449a, miR–668), and five with Anxa2 (miR–542–3p, miR–20a–5p, miR–19a, miR–106–5p, miR–93–5p) (Figure 1D and Figure 3).

Notably, six of the hnRNP A2b1-associated miRNAs (let–7g, let–7d, miR–20a–5p, miR–709, miR–106–5p and miR–93–5p) showed decreased expression in EVs. This depletion is consistent with the reduced hnRNP A2b1 protein levels observed in the EVs from 3–Gy irradiated BMCs. Crucially, five of these targets (let–7g, let–7d, miR–20a–5p, miR–709, and miR–93–5p) were increased in BMCs while decreased in EVs, placing them in the previously defined “cell-retained” or “EV-depleted” group. Overall, this alteration, where cellular abundance does not lead to EV export, suggests that EV loading is an active, protein-dependent process that is disrupted, possibly when the concentration of the suggested transporter protein, hnRNP A2b1, decreases in EVs.

### 2.4. hnRNP A2b1 Binds miRNAs Involved in Bone Marrow Radiation Response and Transports Them into BM–EVs

**Figure 4:**
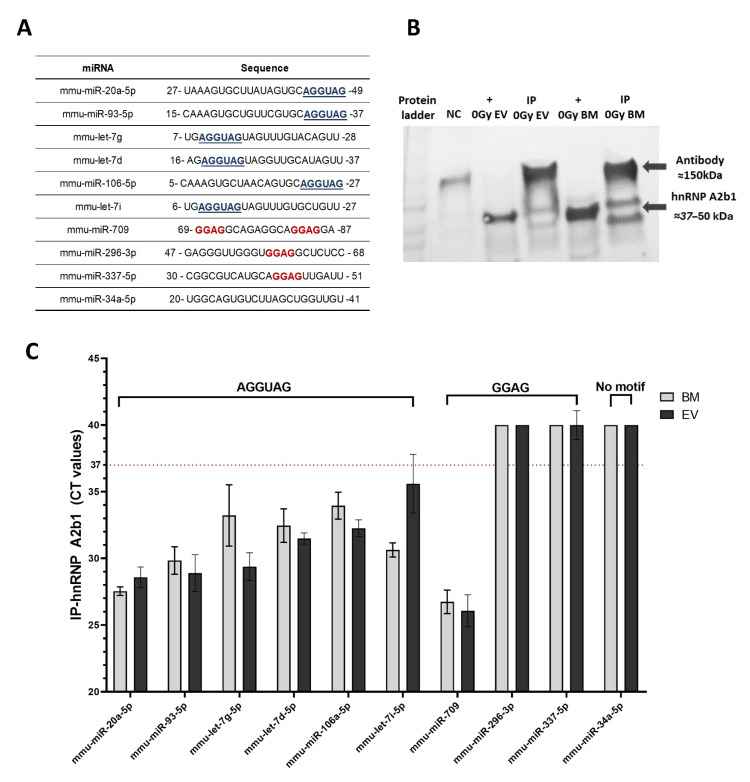
hnRNP A2b1 binds BM and BM–EV miRNAs, showing a preference for the AGGUAG motif. (**A**): Name, sequence, and hnRNP A2b1 recognition motif of miRNAs assayed by RT-qPCR in the hnRNP A2b1 immunoprecipitates of BM and bone BM-EV. (**B**): Western blot detection of immunoprecipitated hnRNP A2b1 protein. NC represents the negative control, where IP was performed using PBS instead of BM or BM-EV samples, confirming that the hnRNP A2b1 antibody signal does not interfere with the protein signal. +0 Gy EV and +0 Gy BM indicate the presence of hnRNP A2b1 in non-immunoprecipitated samples, while IP 0 Gy EV and IP 0 Gy BM demonstrate the presence of hnRNP A2b1 after immunoprecipitation. Multiple bands are observed for hnRNP A2b1 in the blot, likely resulting from post-translational modifications [12,19]. Replicates are presented in Supplementary Figure S8. (**C**): RT-qPCR detection of miRNAs extracted from immunoprecipitates of BM and BM-EVs. Raw Ct values are shown, with miRNAs having a Ct value ≥ 37 (represented by dashed line) considered undetected and displayed as Ct = 40.

miRNA–protein interactions were predicted through miRNA sequence motif analysis, focusing on the presence of GGAG and AGGUAG motifs within mature miRNAs. As shown in Figure 1C,D, hnRNP A2b1 was associated with 10 of the 15 miRNAs that were significantly, or nearly significantly (miR–709, *p* = 0.052), altered in EVs following 3 Gy irradiation. All these miRNAs contained either the AGGUAG or GGAG motif (Figure 4A).

To confirm that hnRNP A2b1 can recognize and bind these motif-containing miRNAs in vivo and transfer them into EVs, immunoprecipitation (IP) assays were performed (Figure 4B). To verify sequence specificity, miR–34a–5p, which lacks the specific binding motifs, was included as a negative control. miRNA–hnRNP A2b1 complexes were isolated from both BMCs and BM-derived EVs, and the associated miRNAs were quantified by RT–qPCR (Figure 4C).

The interaction between hnRNP A2b1 and the six “EV-depleted” miRNAs (miR–93–5p, miR–106–5p, miR–20a–5p, let–7g, let–7d, and miR–709) was analyzed. Additionally, three motif-containing “EV-enriched” miRNAs (miR–337–5p (GGAG), miR–296 (GGAG), and let–7i (AGGUAG)) were also examined.

Seven miRNAs were detected in both BM and EV protein precipitates. This recovered group comprised all six “EV-depleted” miRNAs and one “EV-enriched” miRNA (let–7i); notably, six of these seven contain the AGGUAG motif, while only one (miR–709) contains the GGAG motif. Conversely, the two miRNAs that could not be recovered from the immunoprecipitates (miR–337–5p and miR–296) both contain the GGAG motif (Figure 4C). These findings suggest that hnRNP A2b1 binds more strongly to AGGUAG motifs than to GGAG motifs in both BMCs and BM–EVs.

Changes in EV-miRNA composition can impact recipient cell functions due to the regulatory nature of miRNAs. To investigate how the altered miRNA cargo in 3 Gy-BM-EVs might influence recipient BMC functioning, and to identify altered regulatory pathways for further studies, we performed a KEGG pathway analysis of all 326 deregulated miRNAs (Figure 1A) and the hnRNP A2b1-associated subset (Figure 1C). The 326 deregulated miRNAs were associated with 178 KEGG pathways (Supplementary Table S2A). KEGG pathways were ranked based on the number of miRNAs involved, associated genes, and pathway significance. This ranking step allowed us to prioritize the most relevant pathways with the strongest combined evidence, revealing that chronic leukemia ranked 5th, acute leukemia ranked 55th, cellular senescence ranked 16th, alongside high-scoring inflammatory pathways (Supplementary Figure S2A). These findings highlight the relevance of the differentially expressed EV-miRNAs in leukemia, cellular senescence, and inflammation-related processes (Figure 1B).

**Figure 5:**
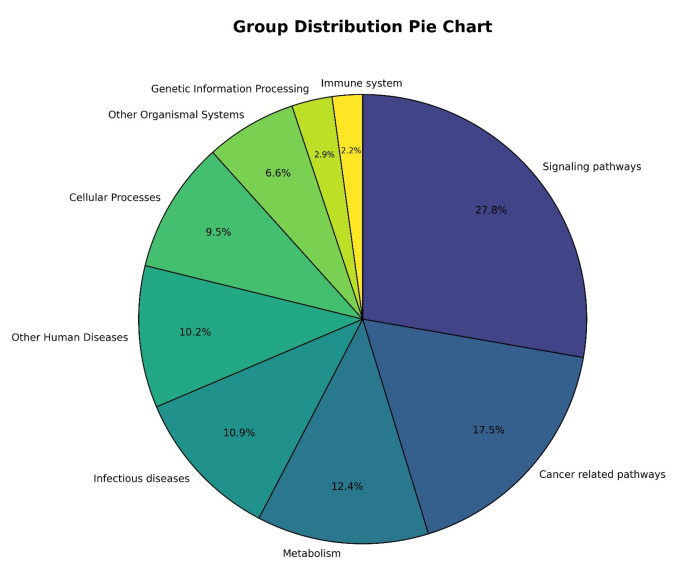
KEGG pathway analysis of 3 Gy-altered miRNAs associated with hnRNP A2b1 through binding motifs. miRNA pathway analysis was performed using DIANA-miRPath v4.0. In the pie chart, significantly deregulated pathways (FDR *p*-value < 0.05) are presented. Pathways were grouped according to their KEGG category and biological function.

Pathway analysis of hnRNP A2b1-associated miRNAs identified 137 significantly deregulated pathways (Supplementary Table S2B), grouped by KEGG category and biological function (Figure 5).

The most highly represented category was signaling pathways (38 pathways, representing 27.7% of all pathways). This group included pathways highly relevant to BM radiation response, such as p53 signaling, signaling pathways regulating pluripotency of stem cells, as well as inflammatory and immune response pathways, including TNF, TGF-beta, and T cell receptor signaling. The second most abundant category was cancer-related pathways (24 pathways, 17.5%), which included, among others, acute and chronic myeloid leukemia, miRNAs in cancer, PD-L1 expression, and PD-1 checkpoint pathway in cancer.

**Figure 6:**
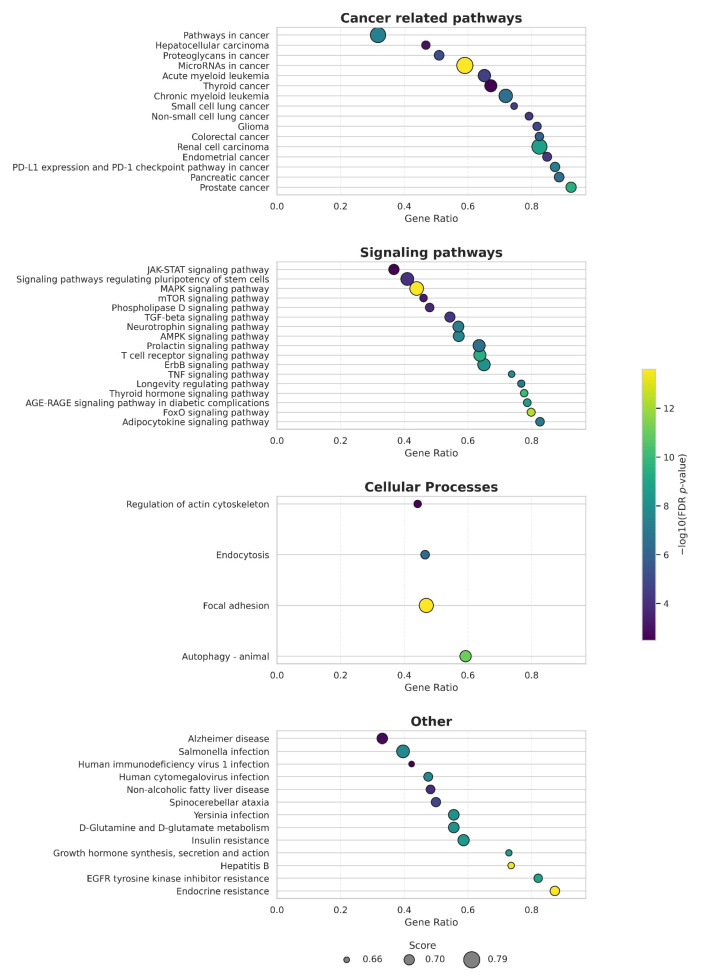
Top 50 hnRNP A2b1-miRNA regulated pathways based on ranked analysis. The top 50 deregulated pathways are presented, grouped by biological function. Pathways were ranked according to a pathway score, which was calculated from three parameters: coverage (targeted genes/total genes in the pathway), number of miRNAs in the pathway, and pathway FDR *p*-value. Bubble size represents the pathway score, reflecting its overall biological relevance, while bubble color indicates the significance level of the pathway (−log10 FDR *p*-value).

Ranking analysis of the 137 KEGG pathways regulated by hnRNP A2b1-associated miRNAs revealed that, consistent with the global miRNA profile, 31 of the top 50 identified pathways were related to cancer and signal transduction (Figure 6). These included pathways associated with aging (longevity regulating pathway), leukemia (acute and chronic myeloid leukemia, and miRNAs in cancer), BM cell development and immune regulation (signaling pathways regulating pluripotency of stem cells, PD-L1 expression and PD-1 checkpoint pathway in cancer, TNF, TGF-beta, JAK-STAT, mTOR, FoxO and T cell receptor signaling pathways).

These results indicate that miRNAs associated with hnRNP A2b1 might be involved in the regulation of pathways relevant for both acute and long-term radiation responses in the BM, including leukemia and immune system regulation.

### 2.5. BM-EVs from Irradiated Mice Induce DNA Damage and Long-Term Senescence in Recipient Bone Marrow

**Figure 7:**
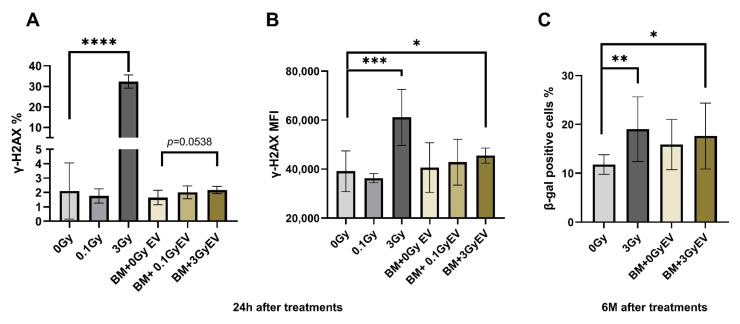
3Gy BM-derived EVs induce DSB and cellular senescence in naïve mice. (**A**,**B**) Flow cytometric analysis of the BM from directly irradiated or EV-treated mice, 24 h after treatment, stained with an anti-γ-H2AX antibody. The total percentage of γ-H2AX positive cells (**A**) and their median fluorescence intensity (MFI) (**B**) are shown. Data are presented as mean ± SD from at least three independent experiments. (**C**) Flow cytometric analysis of the BM from directly irradiated or EV-treated mice, 6 months after treatment, after staining with the Cellular Senescence Detection Kit (SPiDER-β-Gal). The total percentage of SPiDER-β-Gal-positive cells is shown. Data are presented as mean ± SD from 9–12 mice across independent experiments. Statistical significance was determined using Welch’s ANOVA followed by the Welch post hoc test. The difference was considered statistically significant when *p* < 0.05. Significance levels are denoted by asterisks: * for *p* < 0.05, ** for *p* ≤ 0.01, *** for *p* ≤ 0.001, and **** for *p* ≤ 0.0001.

To demonstrate the biological impact of EVs and the functional relevance of the identified KEGG pathways possibly altered by EV-miRNAs, DNA double-strand breaks (DSBs)—a hallmark of radiation damage—were assessed. To this end, γ-H2AX levels were measured in the total BM of both directly irradiated and EV-treated (bystander) mice, 24 h after treatment (Figure 7).

Direct irradiation significantly increased both the proportion of γ-H2AX positive cells and the protein’s median fluorescence intensity (MFI) (15-fold and 1.6-fold, respectively; *p* < 0.001), whereas low-dose (0.1 Gy) exposure induced no significant changes.

In recipient “bystander” animals treated with EVs, the percentage of γ-H2AX-positive cells did not differ significantly from the 0 Gy control. However, comparing 3 Gy EV-treated animals with those receiving 0 Gy EVs revealed a marginal elevation in the γ-H2AX population (1.3-fold change; *p* = 0.0538) (Figure 7A). Furthermore, γ-H2AX MFI analysis confirmed that 3 Gy EV treatment significantly enhanced the DNA damage signal intensity compared to the 0 Gy irradiated control (1.2-fold change; *p* = 0.0353) (Figure 7B).

To evaluate the long-term consequences of IR and to determine whether persistent alterations can be induced by EVs in the BM, cellular senescence was assessed six months after treatment (Figure 7C). For this purpose, the SPiDER-β-Gal detection kit was used as a highly sensitive method to identify altered lysosomal β-galactosidase activity, a well-established biological hallmark of senescent cells. Since significant acute alterations were only observed at higher doses, long-term analysis focused on the 3 Gy and 3 Gy EV groups.

Six months after treatment, the number of β-Gal positive cells was significantly elevated following both 3 Gy direct irradiation (1.6-fold; *p* = 0.0107) and 3 Gy EV treatment (1.5-fold; *p* = 0.0361) (Figure 7C). Notably, senescence levels induced by 3 Gy direct irradiation and 3 Gy EV treatment were highly comparable, showing no statistically significant difference between the two treatments.

While 0 Gy EV treatment showed an upward trend in β-Gal positivity, this effect did not reach statistical significance.

Overall, these results indicate that EVs derived from irradiated BM can transmit radiation-like biological effects to recipient mice, inducing both acute DNA damage and persistent cellular senescence. These findings underscore the functional significance of the actively reshaped EV cargo, particularly following high-dose exposure.

## 3. Discussion

EVs are involved in the regulation of the BM microenvironment [20], partly through the transfer of their miRNA cargo [21,22]. Since IR influences their cargo, EVs might be important vehicles for altering intercellular communication in the BM following IR and might be partly responsible for IR-induced changes in the BM microenvironment. Therefore, one of our main objectives was to compare the miRNA profiles of BMC and BM-derived EVs after IR, and to investigate potential IR effects on the selective miRNA packaging in the EVs.

Previously, we investigated IR effects on the miRNA content of BM-derived EVs 24 h after 3 Gy irradiation [9]. The raw data from our previous work are deposited in the STORE database (DOI:10.20348/STOREDB/1176/1255). In our current study, we identified that among the 326 deregulated miRNAs from BM-derived EVs of mice irradiated with 3 Gy, 47% (154) were related to leukemia or cellular processes involved in leukemogenesis, such as cellular senescence and inflammation. Therefore, miRNAs associated with these three pathways (AML, senescence, and inflammation) were selected for further analysis.

The comparative analysis of miRNA expression in BMCs and BM-derived EVs revealed distinct patterns following irradiation, indicating that IR actively modulates EV-miRNA cargo. While low-dose (0.1 Gy) irradiation had a negligible effect, 3 Gy exposure caused widespread deregulation. In BMCs, the response was predominantly upregulation (observed in 10 of the 12 deregulated miRNAs with RT-qPCR). In contrast, the BM-EV profile did not reflect this cellular response, as most deregulated miRNAs (60%) showed decreased expression. Based on this disparity, we identified distinct packaging categories: a substantial “cell-retained” (or “EV-depleted”) group, which was elevated in cells but decreased in EVs, and conversely, “EV-enriched” miRNAs that increased specifically in vesicles without cellular elevation. Only a minority of the validated miRNAs (17%, 4 out of 24) changed in the same direction in both BM and BM-derived EV. Collectively, these data indicate that IR-induced changes in BM-derived EV miRNA content are the result of an active sorting mechanism. These findings align with the literature; it has been reported that the EV miRNA profile is altered after IR [9,23] as a result of active sorting mechanisms [12,14,24].

To better understand how EV miRNA profiles are regulated, we focused on the role of RNA-binding proteins, which have been reported to actively shape EV RNA cargo [11,12,13,14,25,26,27,28,29,30,31,32,33,34,35,36,37,38,39,40,41,42,43,44,45]. When analyzing miRNA-RBP interactions of the significantly deregulated miRNAs in BM-derived EVs from irradiated mice involved in the three key processes (AML, senescence, and inflammation), 71% of these interactions involved three RBPs (hnRNP A2b1, hnRNP Q, and Anxa2). In addition, these proteins play essential roles in BM function [46,47,48]. They contribute to maintaining the self-renewal capacity of hematopoietic stem cells [46,47], regulate the BM microenvironment [49], and influence cell proliferation and cell-cycle progression [50]. They are also involved in radiation responses, including the DNA-damage response [19,51,52,53], apoptosis [49,54], cell-cycle control, and genome maintenance [50].

hnRNPs are a large family of RNA-binding proteins that play key roles in various posttranscriptional regulatory processes, including alternative splicing, RNA stabilization, degradation, and translation [55]. Owing to their role in regulating gene expression and multiple cellular processes, hnRNP dysregulation has been linked to various cancers, including acute myeloid leukemia (AML) [56].

hnRNP Q plays a critical role in BM function, particularly in maintaining the self-renewal capacity of hematopoietic stem cells [46,47]. It also regulates inflammation [57] and p53-dependent apoptosis [49], both of which are crucial for cellular responses to IR.

In our study, hnRNP Q expression increased following exposure to 3 Gy IR in both BM and BM-derived EVs, with a more pronounced increase observed in the EVs. These findings align with those of Shender et al., who showed elevated hnRNP Q levels in EVs following cellular stress induced by the DNA-damaging chemotherapeutic agent cisplatin [58]. Although we did not observe cytoplasmic relocalization of hnRNP Q following IR, other in vitro studies showed stress-induced cytoplasmic relocalization of the protein [58], possibly accounting for the elevated levels in EVs [58]. These differences in findings may reflect several factors: our in vivo approach analyzed a heterogeneous mixture of BMCs rather than single-cell lines; the stressors in previous studies were chemical agents or heat shock, rather than IR.

Twenty-nine miRNAs were predicted to interact with hnRNP Q through the presence of GGCU/A motifs. Among these, 13 were associated with AML, cellular senescence, and inflammation. HnRNP Q transferred via EVs was shown to initiate proteomic changes in EV-recipient cells, particularly in processes related to cell cycle regulation and DNA repair [58]. The exact mechanisms by which hnRNP Q influences the proteome of EV-recipient cells remain unclear, leaving room for the potential involvement of miRNAs carried by hnRNP Q. This hypothesis is supported by our current findings, where 35% of hnRNP Q-associated miRNAs (10/29) are linked to cellular senescence and thereby cell cycle regulation. Further evidence comes from our previous study [9], where we demonstrated that EVs derived from irradiated mouse BMCs can alter the proteome of nonirradiated BMCs, including changes in cell cycle regulation. This effect was partially mediated by the transfer of miRNAs from irradiated to nonirradiated cells via EVs.

hnRNP A2b1 refers to the closely related proteins hnRNP A2 and hnRNP B1, which differ by just 12 amino acids and are referred to as hnRNP A2b1 or hnRNP A2/B1 in the literature [59]. This protein is widely expressed in many cell types, and its deregulation is associated with various cancer types [60], such as AML [60]. hnRNP A2b1 plays a significant role in the BM by regulating numerous biological processes, including cell cycle control, cell proliferation, inflammation, and genome maintenance [50].

In our study, hnRNP A2b1 concentration decreased in EVs and increased in BMCs 24 h after 3 Gy irradiation, whereas 0.1 Gy IR had no effect. Confocal microscopy and Western blotting demonstrated nuclear relocalization of the protein. This finding aligns with studies showing stress-induced hnRNP A2b1 relocalization [13] and nuclear accumulation following DSB-inducing chemotherapy [14]. The interaction between hnRNP A2b1 and γ-H2AX [51], and the association with DSB hotspots [53], support the hypothesis that hnRNP A2b1 relocalization into the nucleus is a consequence of the elevated DSB formation observed in the BM. Moreover, hnRNP A2b1 is involved in the DNA damage response, negatively impacting DSB repair by inhibiting both homologous recombination and nonhomologous end joining [19,53]. This hypothesis also explains why no nuclear relocalization was observed after 0.1 Gy irradiation, where no significant DNA damage can be detected [15].

To investigate the role of RNA-binding proteins in IR-induced EV-miRNA cargo, hnRNP A2b1 was selected for further analysis. We focused on this protein because it exhibited strong deregulation and distinct concentration changes between BMCs and EVs following 3 Gy irradiation, and most qPCR-validated deregulated miRNAs (10 out of 15) were associated with hnRNP A2b1, making it a suitable model to study selective miRNA packaging. Out of the 10 EV-deregulated miRNAs associated with hnRNP A2b1, six showed concentration changes that mirrored those of the protein (“EV-depleted” miRNAs), suggesting the involvement of an RBP-dependent sorting mechanism. In contrast, four miRNAs changed differently in EVs compared to the EV protein levels, implying alternative loading mechanisms, possibly via multiple recognition motifs or RBP-independent pathways [61].

Immunoprecipitation confirmed sequence-specific binding of hnRNP A2b1 to miRNAs, as only motif-containing miRNAs were co-precipitated with the protein. Binding appeared stronger for AGGUAG motifs compared to GGAG motifs, as all AGGUAG-containing miRNAs were precipitated, whereas only one of three GGAG-containing miRNAs was detected. This difference may reflect a requirement for specific post-translational modifications (PTMs) for GGAG motif recognition. Although PTMs were not directly investigated in this study, multiple bands observed in hnRNP A2b1 blots suggest the presence of distinct PTM forms with different molecular weights [12,19]. Regarding motif recognition, it has been reported that SUMOylation is necessary for GGAG recognition [12], and O-GlcNAcylation can influence hnRNP A2b1–miRNA interactions, including miR-93, which carries an AGGUAG motif [13].

Similar to previous results [12,62], our data demonstrate that hnRNP A2b1 is not only responsible for miRNA transport into EVs but is itself possibly packaged into the EVs, and remains bound to miRNAs after EV incorporation. These findings indicate that hnRNP A2b1 mediates selective miRNA loading into EVs in a motif- and potentially PTM-dependent manner while maintaining miRNA association post-packaging.

It should be noted that protein–miRNA binding assays were performed only on 0 Gy samples and not following irradiation, as our primary goal was to map the changes in the EV-miRNA profile itself. Based on our results, the putative transport protein, hnRNP A2b1, almost entirely disappeared from the EVs following 3 Gy irradiation. Therefore, the observed decrease in specific miRNA levels is more likely explained by the loss of the carrier protein from the vesicles rather than a change in the internal binding dynamics between the protein and miRNAs.

Since the role of hnRNP A2b1 in BM is not well studied, we predicted the functions of its associated miRNAs and their potential involvement in the radiation response of BMCs. Our pathway analysis of hnRNP A2b1-associated miRNAs revealed that miRNAs deregulated following 3 Gy irradiation are involved in a broad range of signaling and regulatory networks relevant to BM radiation response. Several of these pathways (such as p53, T-cell receptor, and TNF signaling, regulation of stem-cell pluripotency, DNA metabolism and repair pathways, and cancer-related pathways, including acute and chronic myeloid leukemia) were significantly altered based on proteome analysis of BMCs treated with EVs derived from 0 Gy-, 0.1 Gy-, and 3 Gy-irradiated cells, as shown in our previous study [9]. These results align with the literature, as hnRNP A2b1 has been reported in various cell types to be involved in regulating cell proliferation, inflammation [50], and DNA damage responses [19,52,53]. Furthermore, deregulated expression of the protein was also associated with AML progression [60].

In the current study, only the EV-miRNA profile was analyzed, and biological alterations were predicted based on the altered miRNome. However, as reported in our previous work [9], only a portion of the BM bystander responses can be linked to miRNAs; other bioactive EV cargo components, such as lipids, proteins, DNA, mRNA, and long non-coding RNAs [63], likely also play a role in mediating radiation-induced bystander effects.

The capability of EVs to induce acute and long-term radiation responses was demonstrated by measuring DNA damage and cellular senescence after EV treatment. In both assays, only 3 Gy BM-derived EVs were able to induce significant alterations. The overall percentage of γ-H2AX-positive cells in the BM did not increase significantly following EV treatment, but protein fluorescence intensity did, suggesting a minor but detectable activation of the DNA damage response pathway. Given the heterogeneous cellular composition of the BM, this change might also reflect induction of the DNA damage response only in particular, small cellular subpopulations.

This possibility of a selective impact is consistent with the fact that different BMC subpopulations exhibit distinct radiosensitivity [8,15] and aligns with the literature showing that various cell types possess varying affinities for EV uptake [64,65].

Finally, we demonstrated that both high-dose irradiation and 3 Gy-derived EVs can induce long-term alterations in the BM, such as cellular senescence. Given that 3 Gy irradiation is known to increase leukemia incidence in CBA/Ca mice [66], it would be interesting to assess how EV-induced DNA damage and senescence might contribute to radiation-induced leukemogenesis.

Our findings suggest a qualitative shift between low- and high-dose radiation responses, reflected in both the DNA damage response patterns and the EV miRNA profiles, rather than a mere dose-dependent quantitative difference. The sensitivity of the analytical platforms used across our studies, including the qPCR used here and the mass spectrometry employed previously [9], supports the conclusion that effects observed at 0.1 Gy reflect different mechanisms rather than a failure to reach detection thresholds. This aligns with the literature [67,68,69], and with our previous proteomic study [9], where low-dose (0.1 Gy) irradiation primarily deregulated proteins associated with mitochondrial processes, oxidative stress, and inflammatory pathways, whereas 3 Gy irradiation and EVs from 3 Gy irradiated cells triggered DNA damage response mechanisms, such as histone deregulation and chromatin remodeling, as well as immune system-related alterations. In the current study, miR–28–5p, a miRNA involved in immune regulation [70], was the only miRNA significantly altered after low-dose irradiation, supporting the role of low-dose irradiation in immune system regulation. Although inflammatory markers were not investigated in this study, our previous work demonstrated that both low- and high-dose IR and EVs can affect proteins involved in inflammatory pathways [9,18]. IR and BM-EVs induced blood-protein alterations [18], and altered the proportions of immune cells in the spleen [15].

It should be noted that one limitation of this study is the use of a murine model. While the evolutionary conservation of fundamental processes—such as DNA damage and cellular senescence—provides a basic mechanistic framework, interspecies differences inherently constrain direct translation to humans. Therefore, further studies exploring radiation-induced bone marrow alterations, including leukemia development, and using human samples, are needed to validate these results.

## 4. Materials and Methods

### 4.1. Murine Model and Irradiation

Nine- to twelve-week-old CBA/Ca male mice were used for the experiments. The mice were kept and handled in accordance with Hungarian animal welfare laws and European 2010/63/EU directives and regulations. The animal studies were approved, and permission was obtained from the Budapest and Pest County Administration Office Food Chain Safety and Animal Health Board (ethical permission: PE/EA/00139–4/2022).

The mice, which were randomly selected from different litters, were subjected to total body irradiation at doses of 0.1 Gy or 3 Gy, with a dose rate of 5.3 cGy/min and 87.5 cGy/min, respectively. The control mice were sham-irradiated at 0 Gy. For irradiation, the X-RAD 225/Xli X-ray source (Precision X-ray, North Branford, CT, USA) was used. Twenty-four hours after irradiation, the mice were sacrificed via intraperitoneal injection of Euthanimal 40% solution (Alfasan, JaWoerden, The Netherlands).

### 4.2. Sample Collection

BMCs were flushed from the femurs and tibias of the mice with ice-cold PBS. After a single-cell suspension was created through the mechanical disruption of the BM tissue, the cells were pelleted by centrifugation at 500× *g* at 4 °C for 10 min. The supernatant was collected for the isolation of BM-derived extracellular vesicles. The pelleted BMCs were resuspended in ice-cold phosphate-buffered saline (PBS), filtered through a 100 µm cell strainer (VWR, Radnor, PA, USA), and quantified via a Bürker Chamber. Pelleted BMCs were immediately utilized for subsequent analyses (immunocytochemistry, immunoprecipitation, subcellular fractionation, and flow cytometric analysis) or stored at −80 °C with or without RLT Plus RNA isolation buffer (Qiagen, Hilden, Germany).

### 4.3. Extracellular Vesicle Isolation and Characterization

BM-derived EVs were isolated from the pooled BM supernatants of three mice via the ExoQuick-TC system (System Biosciences, Palo Alto, CA, USA) as described previously [15]. In brief, a 1:5 ratio of ExoQuick-TC reagent to the supernatant was incubated overnight at 4 °C. EVs were pelleted by centrifugation at 1500× *g* at 4 °C for 30 minutes and resuspended in 280 µL of 0.1 µm filtered cold PBS. To enhance EV purity, PD SpinTrap G-25 desalting columns (GE Healthcare, Marlborough, MA, USA) were used. Native EV samples and EV samples mixed with RLT Plus RNA isolation buffer were stored at −80 °C until use.

EVs isolated from murine BM with the ExoQuick-TC system were characterized in our previous works [8,9], based on their size, concentration, morphology, and protein content. Here, the presence of specific EV markers was detected via Western blotting, and particle size was determined via tunable resistive pulse sensing (TRPS) (Supplementary Figure S1).

### 4.4. Immunocytochemistry and Image Analysis

Round glass coverslips were positioned at the bottom of 24-well plates, onto which freshly isolated BMCs (2 × 10^5^ BMCs/well in 500 µL of PBS) were pelleted through centrifugation at 250× *g* at room temperature (RT) for 2 min. The cells were fixed with 2% paraformaldehyde (PFA) at RT for 5 min. Subsequently, the cells were washed twice with PBS containing 50 mmol/L glycine. To label the cell membrane, Alexa Fluor 488-conjugated wheat germ agglutinin (WGA) (Thermo Fisher Scientific, Waltham, MA, USA) was diluted in PBS (1:500) and incubated with the cells for 30 min at RT. The cells were permeabilized with 500 µL of PBS/well containing 0.25% Triton X-100 and 0.1% glycine at RT for 10 min. The cells were incubated with blocking buffer (3% BSA in PBS) at RT for 30 min, followed by an overnight incubation at 4 °C with the following primary antibodies: anti-hnRNP A2b1 (ab31645, Abcam, Cambridge, UK) and anti-hnRNP Q (R5653, Merck, Rahway, NJ, USA), followed by a 30 min RT incubation with the following secondary antibodies: goat anti-mouse IgG H&L (A10524, Thermo Fisher Scientific, Waltham, MA, USA, dilution: 1:750) and goat anti-rabbit IgG H&L (AB150078, Abcam, Cambridge, UK, dilution: 1:500). Coverslips were mounted on slides with 4′,6-diamidino-2-phenylindole (DAPI)-containing mounting medium (ab104139; Abcam, Cambridge, UK).

Fluorescent labeling was visualized with a Leica TCS SP8 Lightning Confocal Laser Scanning microscope and LASX software Version 3.5.7 (Leica, Germany). Images were captured in three sequences: sequence 1, DAPI (PMT detector) +Cy5 hybrid detector; sequence 2, Alexa Fluor 555 hybrid detector; and sequence 3, CF488 hybrid detector.

To quantify the fluorescence intensity and determine the cellular localization of the labeled proteins, ImageJ software, the “Intensity Ratio Nuclei Cytoplasm” tool, was used [71,72]. The fluorescence intensity of the labeled proteins (hnRNP A2b1 and hnRNP Q) was measured in both the nucleus (indicated by DAPI staining) and the entire cell (indicated by WGA plasma membrane staining). The measured fluorescence intensity was normalized to the area to obtain an average fluorescence intensity. Cytoplasmic values (intensity and area) were determined by subtracting the nuclear signal from the entire cell signal. To obtain the nuclear/cytoplasmic ratio, the average nuclear fluorescence intensity was divided by the average cytoplasmic fluorescence intensity. To be included in the analysis, the cells had to meet specific criteria: a clearly visible and intact cytoplasmic membrane labeled with WGA, an intact nucleus labeled with DAPI, and the entire cell had to be visible in the image.

### 4.5. Preparation of Nuclear and Cytoplasm-Enriched Extracts

For subcellular fractionation, freshly isolated BMCs (8 × 10^6^) were centrifuged at 500× *g* at 4 °C for 5 min. The resulting pellet was resuspended in pre-extraction buffer (ab113474; Abcam, Cambridge, UK) and incubated on ice for 5 min. To achieve complete cell lysis, the solution was transferred into a Dounce homogenizer and subjected to 25 iterations of up-and-down passes of the pestle. Nuclear-enriched fractions were pelleted by centrifugation at 1000× *g* at 4 °C for 5 min. The supernatant, representing the cytoplasm-enriched fraction, was collected and further clarified by centrifugation at 15,000× *g* at 4 °C for 3 min to remove residual debris; the resulting supernatant was stored at −80 °C. The nuclear-enriched pellet was washed in 100 µL of ice-cold PBS and subsequently lysed using radioimmunoprecipitation assay (RIPA) lysis buffer (RIPA: 50 mM Tris-HCl, pH 8; 150 mM NaCl; 1% NP-40; 0.5% sodium deoxycholate; 0.1% SDS) supplemented with Halt protease inhibitor (78425, Thermo Fisher Scientific, Waltham, MA, USA) The nuclear-enriched fraction was stored at −80 °C for later use.

The protein content of the nuclear- and cytoplasm-enriched fractions was assessed through Western blotting.

### 4.6. Western Blot and Protein Quantification

To identify and quantify specific proteins, Western blot (WB) analysis was conducted as previously described [9]. Briefly, whole cells (3 × 10^6^) and EVs were lysed with RIPA lysis buffer supplemented with Halt protease inhibitor (Thermo Fisher Scientific, Waltham, MA, USA), and the protein concentration was determined via the bicinchoninic acid (BCA) assay (Pierce™ BCA Protein Assay Kit, Thermo Fisher Scientific, Waltham, MA, USA). Forty micrograms of protein were precipitated with trichloroacetic acid (TCA), pelleted by centrifugation at 3800× *g* and 4 °C for 5 min, and then washed twice with ice-cold acetone. Protein precipitates were resuspended in 10 µL of PBS, diluted 1:2 in Laemmli buffer (Bio-Rad Hercules, CA, USA) supplemented with β-mercaptoethanol, and boiled at 95 °C for 5 min. Gel electrophoresis was performed via sodium dodecyl sulfate-polyacrylamide gel electrophoresis (SDS-PAGE) with 10% or 4–20% Mini-PROTEAN^®^ TGX Stain-Free polyacrylamide gels (Bio-Rad, Hercules, CA, USA), and the proteins were transferred overnight onto a polyvinylidene fluoride (PVDF) membrane (Thermo Fisher Scientific, Waltham, MA, USA). The PVDF membrane was blocked with 3% BSA-TBST buffer for 30 min, then incubated with primary antibodies for 2 h at room temperature with gentle shaking, and with secondary antibodies for 1 h at room temperature with gentle shaking. Visualization of protein bands was achieved via the chromogenic method with 3,3′-diaminobenzidine substrate (Pierce™ DAB Substrate Kit, Thermo Fisher Scientific, Waltham, MA, USA). Blots were captured using an Alliance™ Q9 (UVITEC Ltd., Cambridge, UK). For protein quantification, the density of the protein bands was recorded and analyzed via ImageJ software (Image Processing and Analysis in Java, National Institutes of Health, Bethesda, MD, USA).

For WB analysis, the following antibodies were used: anti-calnexin antibody (ab22595), anti-TSG101 antibody (ab125011), anti-hnRNP A2b1 antibody (ab31645), anti-hnRNP Q antibody (ab184946), anti-annexin-2 antibody (ab235939), anti-annexin antibody (ab182646), goat anti-rabbit IgG H&L (HRP) (ab6721), anti-beta actin antibody (PA1183), and anti-CD9 antibody (MA5–31980). The latter two antibodies were purchased from Thermo Fisher Scientific (Waltham, MA, USA), and the other antibodies were from Abcam (Abcam, Cambridge, UK). As molecular weight markers, the following standards were used: Prestained Protein Ladder—broad molecular weight (ab116028, Abcam, Cambridge, UK), Spectra™ Multicolor Broad Range Protein Ladder (26634, Thermo Fisher Scientific, Waltham, MA, USA), and Precision Plus Protein Dual Color Standards (Bio-Rad, Hercules, CA, USA).

### 4.7. Immunoprecipitation

An immunoprecipitation (IP) assay was performed with freshly isolated BMCs and BM-EVs via a Pierce classic IP kit (Thermo Fisher Scientific, Waltham, MA, USA). First, the samples were lysed in IP lysis/wash buffer supplemented with Halt protease inhibitor (Thermo Fisher Scientific, Waltham, MA, USA). A total of 1000 µg wet cell pellets or 700 µg EVs were used. To quantify EVs, the surface proteins of intact vesicles were measured with a Bradford assay (Thermo Fisher Scientific, Waltham, MA, USA). Following sample lysis, the protein concentration was determined via the BCA assay, and 500 µg of protein lysate was used for IP.

Sample lysates were precleared with control agarose beads, where 40 µL of control agarose resin slurry was incubated with 500 µg of protein for 1 h at 4 °C. Control beads were removed from the samples through spin column centrifugation. The cleared samples were mixed with hnRNP A2b1 primary antibody (ab31645, Abcam, Cambridge, UK), diluted to a final volume of 500 µL with IP lysis/wash buffer, and incubated at 4 °C overnight. To capture the formed immune complex, 20 µL of protein A/G plus agarose was added to the antibody–lysate mixture. The mixture was incubated on ice with gentle shaking for 2 h. The samples were divided into two parts, transferred into two separate spin columns, and centrifuged at 1000× *g* at 4 °C for 30 s. Only proteins captured by the antibody remained in the spin column after the centrifugation step. The samples were subsequently washed twice with lysis/wash buffer. One-half of each sample was used for WB validation of the IP results, while the other half was used for microRNA (miRNA) isolation. For IP validation, the spin column was washed with conditioning buffer, 60 µL of nonreducing lane marker sample buffer supplemented with dithiothreitol (DTT) was added to the column, and the sample was boiled twice at 100 °C for 5 min. To collect the eluate containing the immune complex, the column was centrifuged at 1000× *g* and 4 °C for 1 min. The eluted sample was then loaded onto a 4–20% polyacrylamide gel and processed further, following the procedures described in the Western blot section.

For miRNA isolation, 700 µL of QIAzol lysis reagent was added to the second spin column and incubated at room temperature for 5 min. The RNA content was eluted via centrifugation at 1000× *g* and 4 °C for 1 min, and miRNA isolation was performed as described in detail in the miRNA extraction section.

### 4.8. miRNA Extraction and Quantitative RT-qPCR

For miRNA isolation, total RNA containing miRNA was extracted from both BMCs and BM-EVs previously frozen in RLT buffer. RNA isolation and the PCR process are detailed in our previous works [8,9]. Briefly, the extraction process was performed via the RNeasy Plus Mini Kit (Qiagen, Hilden, Germany) in accordance with the manufacturer’s instructions manufacturer’s Protocol booklet Appendix D). In brief, 5 × 10^6^ BMCs were lysed with RLT buffer and transferred to a genomic DNA eliminator spin column. The resulting flow-through was combined with 100% ethanol and centrifuged in a spin column. The flow-through was subsequently discarded, and the column was washed with RPE buffer. Purified total RNA was eluted with 80 µL of nuclease-free water. For complementary DNA (cDNA) synthesis, 2 µL of RNA (40 ng/µL) was used. cDNA synthesis was carried out by using a miRCURY™ LNA™ miRNA RT Kit (Qiagen, Hilden, Germany) according to the manufacturer’s instructions. For qPCR, Maxima SYBR Green Master Mix (Thermo Fisher Scientific, Waltham, MA, USA) was used. cDNA was diluted 20-fold and assayed in a 10 µL PCR volume using a Rotor-Gene Q real-time PCR cycler (Qiagen, Hilden, Germany). Amplification curves were analyzed via Rotor-Gene Q Series software (software version 2.1.0.9) for both threshold cycle (Ct) and melting curve (Tm) analyses. The delta Ct method was used to detect changes in the concentrations of the assayed miRNAs, where irradiated samples (0.1 Gy or 3 Gy) were compared with the control and sham-irradiated (0 Gy) samples. In terms of quantification, mmu–miR–423–3p served as a normalizer due to its consistent and well-detectable concentration in both BMCs and BM-EVs, which remained unchanged upon irradiation. PCR products with a Ct > 37 were excluded from the analysis.

For the IP samples, the miRNA-enriched RNA fraction was isolated via the miRNeasy mini RNA isolation kit (Qiagen, Hilden, Germany) according to the manufacturer’s instructions. Briefly, samples that were lysed with QIAzol and then eluted from the IP spin column were mixed with chloroform and then centrifuged for 15 min at 12,000× *g* at 4 °C. The aqueous phase was collected, combined with 70% ethanol, and centrifuged in a spin column. The resulting flow-through was mixed with 100% ethanol and centrifuged again. The silica membrane was washed first with RPE buffer and then with 80% ethanol. The miRNA content was eluted in 20 μL of nuclease-free water. For cDNA synthesis, 2 µL of RNA (5 ng/µL) was used; cDNA synthesis and PCR were carried out as described above. After amplification curve analysis, the Tm values were compared with the Tm values registered in the BM and EV samples to ensure that the amplification products were similar. PCR products with a Ct > 37 were considered not detectable.

The following miR primers, all purchased from Qiagen (Hilden, Germany), were used: mmu–miR–302c (GeneGlobe ID—YP00205064), mmu–miR–542–3p (GeneGlobe ID—YP00205444), mmu–miR–19a (GeneGlobe ID—YP00205862), mmu–miR–296–3p (GeneGlobe ID—YP00204393), mmu–miR–539–5p (GeneGlobe ID—YP00205656), mmu–miR–383–5p (GeneGlobe ID—YP00205000), mmu–miR–125b–1–3p (GeneGlobe ID—YP00204400), mmu–miR–668–3p (GeneGlobe ID—YP00205049), mmu–miR–27b–3p (GeneGlobe ID—YP00205915), mmu–miR–377–3p (GeneGlobe ID—YP00204733), mmu–miR–449a (GeneGlobe ID—YP00204481), mmu–miR–34a–5p (GeneGlobe ID—YP00204486), mmu–miR–455–3p (GeneGlobe ID—YP00205432), mmu–let–7d–5p (GeneGlobe ID—YP00204124), mmu–miR–337–5p (GeneGlobe ID—YP00205184), mmu–miR–28–5p (GeneGlobe ID—YP00204322), mmu–let–7g–5p (GeneGlobe ID—YP00204565), mmu–let–7i–5p (GeneGlobe ID—YP00204394), mmu–miR–106a–5p (GeneGlobe ID—YP00205061), mmu–miR–20a–5p (GeneGlobe ID—YP00204292), mmu–miR–129–5p (GeneGlobe ID—YP00204534), mmu–miR–708–5p (GeneGlobe ID—YP00204490), mmu–miR–93–5p (GeneGlobe ID—YP00204715), mmu–miR–709 (GeneGlobe ID—YP00205463), mmu–miR–423–3p (GeneGlobe ID—YP00203906).

### 4.9. Analysis of the Interactions of miRNAs in Bone Marrow-Derived Extracellular Vesicles with RNA-Binding Proteins

Based on the literature data, 15 RNA-binding proteins with known miRNA-binding motifs were selected (Supplementary Table S1). To predict RNA-binding protein and miRNA interactions, the raw data from our previous work (deposited in the STORE database, storedb.org/store_v3/, DOI:10.20348/STOREDB/1176/1255) were used. This dataset contains miRNA profile changes in BM-derived EVs, which were generated via NanoString analysis. This dataset was previously analyzed with a focus on the identification of miRNAs with dose-dependent alterations and their involvement in the development of radiation-induced bystander effects. In the present study, we focused specifically on significantly altered miRNAs in EVs following irradiation with 3 Gy. The sequences of the selected miRNAs were analyzed for the presence of miRNA-binding motifs associated with the 15 selected RNA-binding proteins (Supplementary Table S1). miRNAs were included in the analysis if their levels were significantly altered in 3 Gy EVs compared with 0 Gy EVs, with a Benjamini–Hochberg adjusted *p*-value < 0.05 and a log_2_-fold change (FC) > 1.5 or < −1.5 following ionizing radiation. To identify miRNAs relevant for the BM radiation response, miRNAs associated with cellular senescence, inflammation, and acute myeloid leukemia (AML) were selected from a panel of miRNAs that interact with RNA-binding proteins.

To link significantly altered miRNAs (adjusted *p*-value < 0.05) with processes related to cellular senescence, inflammation, or AML, biological databases such as the Kyoto Encyclopedia of Genes and Genomes (KEGG) [73,74,75] and Gene Ontology (GO) [76,77] were used, along with relevant literature data.

To assess the functions of all significantly deregulated EV-miRNAs, and miRNAs bearing hnRNP A2b1 binding motifs (hnRNP A2b1-related miRNAs), DIANA-miRPath v4.0 [78] was used for KEGG pathway analysis, where miRNA-mRNA targets originated from the experimentally validated database TarBase v8.0 [79], and pathway union was used as the merging method. Pathways were considered significant if their FDR-corrected *p*-value was lower than 0.05. For the ranked analysis, coverage for each KEGG pathway was calculated by dividing the number of targeted genes by the total number of genes in the pathway. Coverage values and the number of associated miRNAs were then min–max normalized to scale values between 0 and 1. FDR *p*-values were inverted and similarly normalized so that more significant pathways received higher values. A combined score for each pathway was calculated as the average of the three normalized metrics—target gene coverage, miRNA count, and FDR significance. Pathways were subsequently ranked based on this combined score, with the highest-scoring pathways considered the most relevant for downstream analyses.

### 4.10. Flow Cytometric Analysis

For senescence detection, 5 × 10^6^ BMCs were analyzed per mouse, using the Cellular Senescence Detection Kit (SPiDER-β-Gal; SG03, Dojindo Laboratories, Kumamoto, Japan).

Pelleted cells were resuspended in 300 µL Bafilomycin A1 working solution and incubated for 1 h at 37 °C in a humidified atmosphere containing 5% CO_2_. Following this, 300 µL SPiDER-β-Gal working solution was added directly to the cell suspension. Cells were then incubated for an additional 30 min under identical conditions (37 °C, 5% CO_2_). After incubation, cells were washed with 2 mL cold PBS and pelleted at 500 × g for 10 min at 4 °C. The cell pellet was then resuspended in 150 µL 1% PFA (paraformaldehyde) solution and incubated overnight at 4 °C prior to analysis.

To detect DNA double-strand breaks, 1 × 10^6^ BMCs were pelleted and resuspended in 100 µL of cold PBS, and 2.5 µL of ViaKrome 638 Fixable Viability Dye (Beckman Coulter Inc., Brea, CA, USA) was added. After a 30 min incubation at 4 °C in the dark, cells were washed with 1 mL of cold PBS and centrifuged at 500× *g* for 10 min at 4 °C. BMCs were then permeabilized for 30 min at RT in the dark using 500 µL of FIX/PERM working solution (1:4 ratio of fixation/permeabilization concentrate to fixation/permeabilization diluent). Following permeabilization, the cells were washed with 1 mL of FIX/PERM wash buffer (1 part Permeabilization buffer to 9 parts cold PBS) and centrifuged again at 500× *g* for 10 min at 4 °C. The resulting pellets were resuspended in 50 µL of PBS supplemented with 1.5 µL of H2A.X (Ser139) (20E3) Rabbit Monoclonal Antibody (Alexa Fluor^®^ 488 Conjugate) (Cell Signaling Technology Inc., Danvers, MA, USA) and incubated for 30 min at RT in the dark. After staining, the cells were washed with 1 mL of FIX/PERM wash buffer and centrifuged (500× *g*, 10 min, 4 °C). Finally, the cells were fixed in 100 µL of 1% PFA for 15 min at 4 °C prior to analysis.

Flow cytometry was performed using a CytoFLEX system (Beckman Coulter, Brea, CA, USA) flow cytometer. Data management and analysis were integrated through CytExpert software (version 2.3.0.84).

### 4.11. Statistical Analysis

Statistical analysis was performed via GraphPad Prism version 7.00 for Windows (GraphPad Software, La Jolla, CA, USA). The data are presented as the mean ± standard deviation (SD). Sample normality and homogeneity were tested using the Shapiro–Wilk test and Levene’s test, respectively. To determine statistical significance, Student’s *t* test or its nonparametric alternative, the Mann-Whitney U test, was used. For multiple-group comparisons in which variances were unequal, Welch’s ANOVA was performed, followed by pairwise post hoc Welch’s tests to determine differences between specific groups.

Data were considered statistically significant if the *p*-value or the Benjamini-Hochberg adjusted *p*-value was lower than 0.05. Significance levels are denoted by asterisks: * for *p* < 0.05, ** for *p* ≤ 0.01, *** for *p* ≤ 0.001, and **** for *p* ≤ 0.0001.

## 5. Conclusions

Our study demonstrates that IR actively reshapes the miRNA landscape of BMCs and their EVs, with selective packaging largely mediated by RBPs, with a main focus on hnRNP A2b1. The differential regulation of miRNA expression between BMCs and EVs, together with motif-dependent binding, indicates that RBPs like hnRNP A2b1 orchestrate the selective export of miRNAs into EVs. This sorting mechanism appears to be dose-dependent, as significant changes were mostly observed after 3 Gy but not after low-dose (0.1 Gy) irradiation. IR-induced nuclear relocalization of hnRNP A2b1 reduces its incorporation into EVs, which parallels the depletion of its associated miRNAs from EV cargo. Sequence-specific interactions, with a preference for the AGGUAG motif, further support a regulated sorting mechanism.

We also demonstrated the functional consequences of EV cargo alterations: EVs derived from 3 Gy-irradiated BMCs acted as mediators of radiation-induced bystander effects and could induce elevated DNA damage as well as long-term cellular senescence in nonirradiated BMCs. Pathway analyses further indicated that hnRNP A2b1-associated miRNAs regulate key BM-related processes, including p53 signaling, stem cell pluripotency, immune modulation, and leukemia-associated pathways. Collectively, these findings highlight the central role of RBPs in shaping EV miRNA cargo and suggest their involvement in radiation-induced bystander processes related to the regulation of acute and long-term hematopoietic homeostasis.

## Data Availability

The miRNA dataset analyzed during the current study is available in the STORE database (storedb.org/store_v3/) under STOREDB: STUDY1176 ID (DOI:10.20348/STOREDB/1176/1255).

## Supplementary Materials

The following supporting information can be downloaded at: https://www.mdpi.com/article/10.3390/ijms27125510/s1.

## Abbreviations

The following abbreviations are used in this manuscript:

AML	Acute myeloid leukemia
Anxa2	Annexin-2
BCA	Bicinchoninic acid
BM	Bone marrow
BMC	Bone marrow cells
BSA	Bovine serum albumin
cDNA	Complementary DNA
Ct	Threshold cycle
DAB	3,3′-diaminobenzidine
DAPI	4′,6-diamidino-2-phenylindole
DSB	DNA double-strand break
DTT	Dithiothreitol
EV	Extracellular vesicle
FC	Fold change
FDR	False discovery rate
GO	Gene Ontology
hnRNP A2b1	Heterogeneous nuclear ribonucleoprotein A2/B1
hnRNP Q	Heterogeneous nuclear ribonucleoprotein Q
HSC	Hematopoietic stem cell
IP	Immunoprecipitation
IR	Ionizing radiation
KEGG	Kyoto Encyclopedia of Genes and Genomes
ME	Microenvironment
MFI	Median fluorescence intensity
miRNA	MicroRNA
PBS	Phosphate-buffered saline
PFA	Paraformaldehyde
PVDF	Polyvinylidene fluoride
RBD	RNA-binding domains
RBP	RNA-binding proteins
RIBE	Radiation-induced bystander effect
RIPA	Radioimmunoprecipitation assay lysis buffer
RT-qPCR	Reverse Transcription Quantitative Polymerase Chain Reaction
RT	Room temperature
SDS-PAGE	Sodium dodecyl sulfate-polyacrylamide gel electrophoresis
SD	Standard deviation
TCA	Trichloroacetic acid
Tm	Melting curve
TSG101	Tumor susceptibility gene 101
WB	Western blot
WGA	Wheat germ agglutinin
β-Gal	β-Galactosidase
