# Supplementary material for: Ionizing Radiation Actively Reshapes Bone Marrow-Derived Extracellular Vesicle MicroRNA Cargo with the Involvement of hnRNP A2b1
Source: Int J Mol Sci. 2026 Jun 18;27(12):5510. doi: 10.3390/ijms27125510 (PMC13299799; doi:10.3390/ijms27125510)
Supplement: Supplementary file 1 [file ijms-27-05510-s001.zip › Supplementary figures 1-8m.pdf]

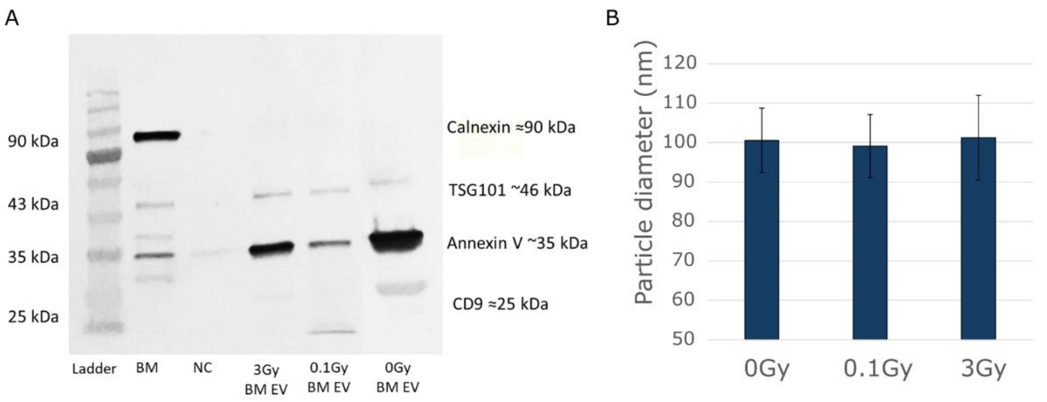

Supplementary Figure 1: Characterization of Bone Marrow-Derived Extracellular Vesicles. **A** Representative Western blot analysis of whole cell lysates and extracellular vesicles isolated from the bone marrow of mice irradiated with the indicated doses of ionizing radiation. As a negative control (NC) EV isolation was carried out using PBS as a starting material. Forty micrograms of protein were loaded on an SDS–polyacrylamide gel. Calnexin was used as a cytoplasmic marker, while TSG101, Annexin5 and CD9 were used as EV markers. Proteins were visualized using secondary antibodies and DAB staining. As amolecular weight marker, Prestained Protein Ladder - Broad molecular weight (ab116028) was used. **B** Size distribution analysis of extracellular vesicle suspensions via TRPS using an NP150 nanopore (measurement range: 70–420 nm). Data represent the mean mode size ± standard deviation (SD) (n=3).

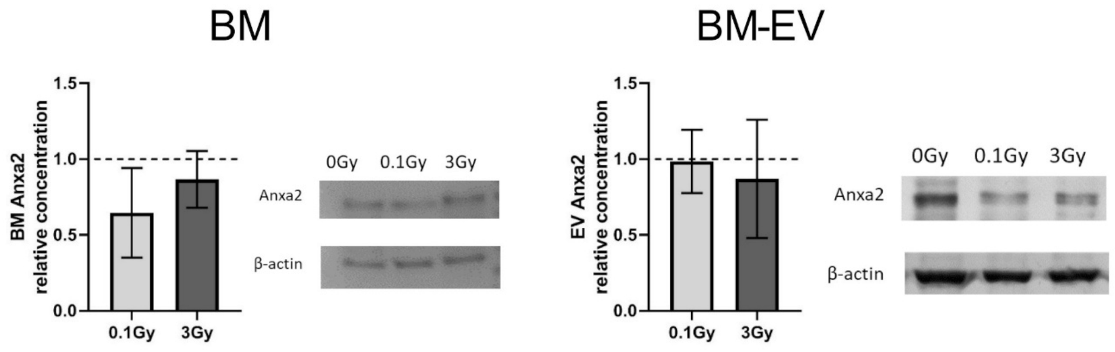

Supplementary Figure 2: Western blot of Anxa2 in bone marrow and EVs 24 hours post-irradiation. Forty micrograms of protein were loaded onto SDS-polyacrylamide gels, stained with anti- anti-Anxa2 primary antibodies, and visualized using secondary antibodies and DAB staining. β-actin was used as a loading control. Quantification was performed using ImageJ software (n=3). For Anxa2 analysis, samples were split (40 μg each) prior to loading and stained separately from β-actin to mitigate high background signal and avoid interference due to similar molecular weights. Bio-Rad Dual Color protein ladder (1610374) was used as a reference.

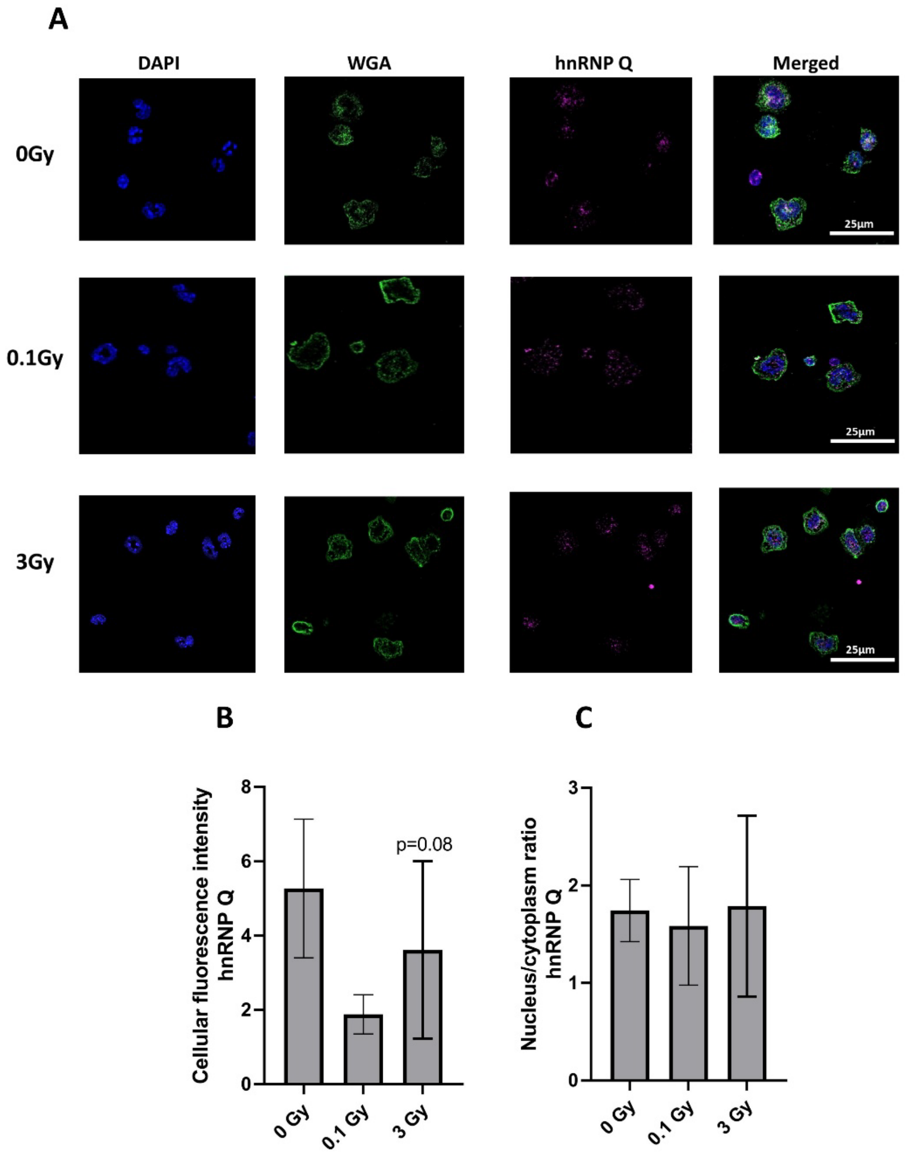

Supplementary Figure 3: Cellular distribution analysis of hnRNP Q with confocal microscopy. Bone marrow cells were stained with Dapi (blue, nucleus), WGA (green, cell membrane), and hnRNP Q specific antibody (purple), fluorescence intensity was calculated with ImageJ (n=3).

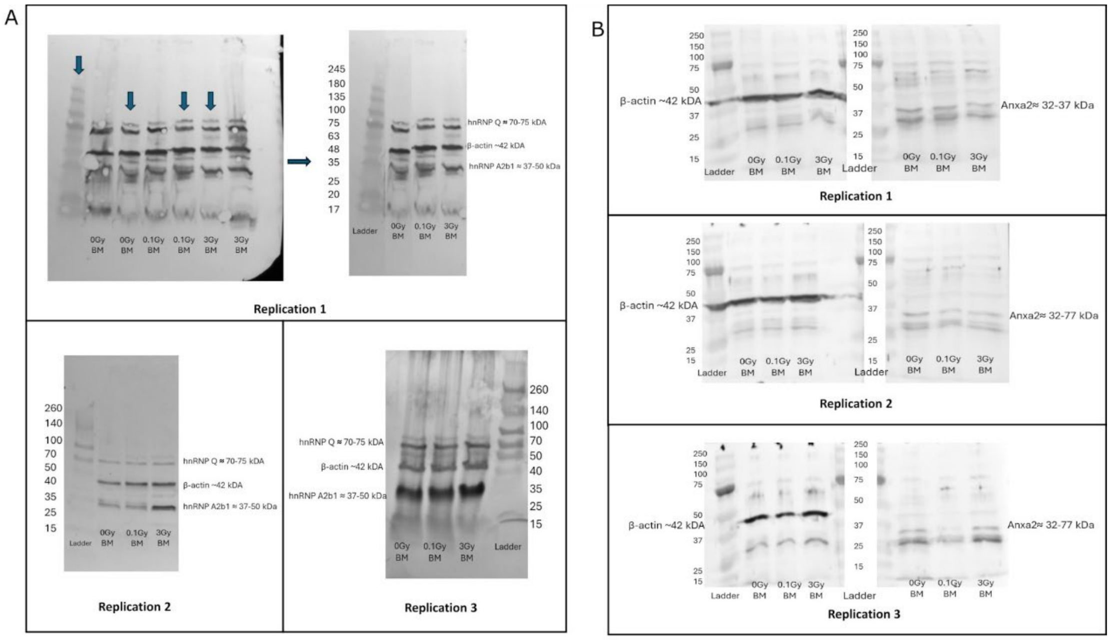

Supplementary Figure 4: Western blot of hnRNP A2B1, hnRNP Q, and Anxa2 in bone marrow 24 hours post-irradiation. Forty micrograms of protein were loaded onto SDS-polyacrylamide gels, stained with anti-hnRNP A2B1, anti-hnRNP Q, or anti-Anxa2 primary antibodies, and visualized using secondary antibodies and DAB staining. β-actin was used as a loading control. Quantification was performed using ImageJ software (n=3). For Anxa2 analysis, samples were split (40 µg each) prior to loading and stained separately from β-actin to mitigate high background signal and avoid interference due to similar molecular weights. The following protein ladders were used: Abcam (ab116028), Thermo Scientific™ (26634), and Bio-Rad (1610374). Figure A shows three Western blots from BM samples for hnRNP A2b1 and hnRNP Q. In the “replication 1” blot in Figure A, lanes indicated by arrows were used for analysis. Figure B shows Western blots from BM samples for annexin A2. For annexin A2, 80 µg of protein per sample was prepared and 40 µg was loaded into each of two separate gel lanes.

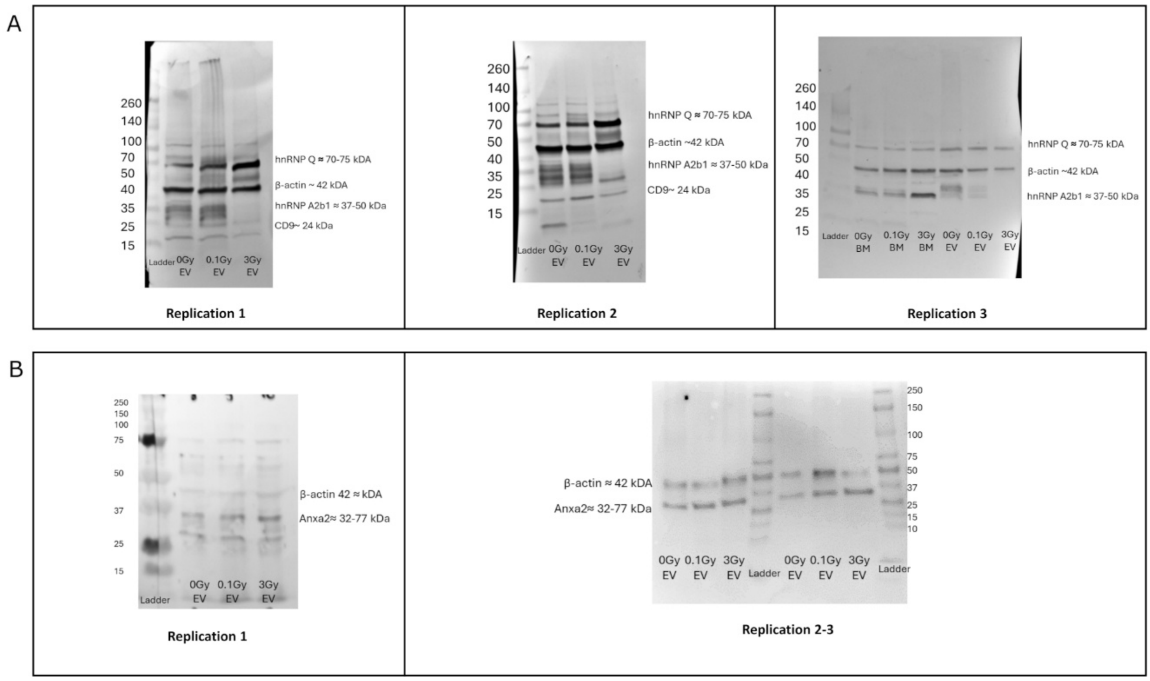

Supplementary Figure 5: Western blot of hnRNP A2B1, hnRNP Q, and Anxa2 in bone marrow-EV 24 hours post-irradiation. Forty micrograms of protein were loaded onto SDS-polyacrylamide gels, stained with anti-hnRNP A2B1, anti-hnRNP Q, or anti-Anxa2 primary antibodies, and visualized using secondary antibodies and DAB staining. β-actin was used as a loading control. Quantification was performed using ImageJ software (n=3). Unlike bone marrow samples, the lower background in EV lysates allowed for the simultaneous staining of Anxa2 and β-actin. The following protein ladders were used: Abcam (ab116028), Thermo Scientific™ (26634), and Bio-Rad (1610374). Figure A shows four Western blots from EV samples for hnRNP A2b1 and hnRNP Q, whereas Figure B shows three Western blots from EV samples for annexin A2.

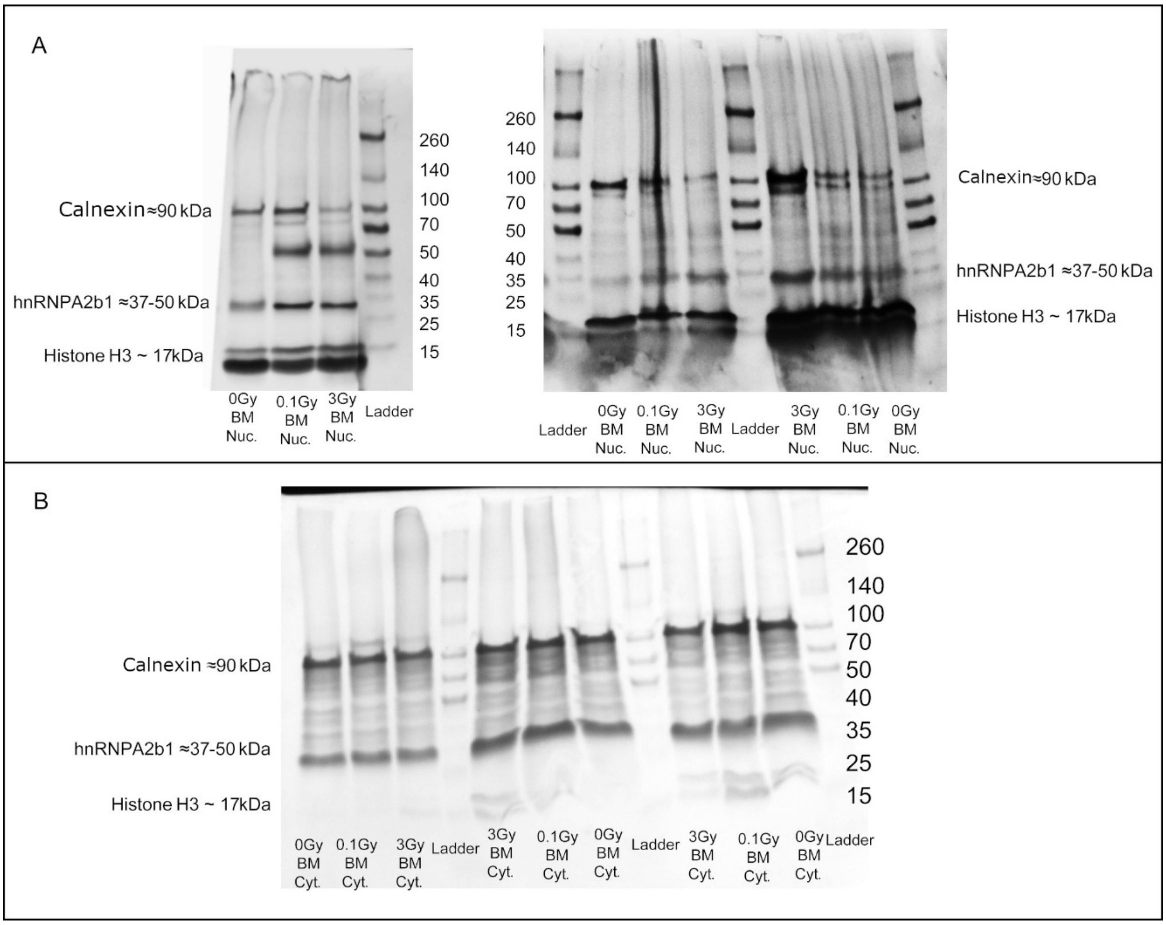

Supplementary Figure 6: Western blot validation of hnRNP A2B1 cellular relocalization 24 hours after irradiation. Nuclear-enriched (A) and cytoplasm-enriched (B) fractions were extracted, and forty micrograms of protein were loaded onto SDS-polyacrylamide gels and stained with anti-hnRNP A2B1 antibodies. Calnexin was used as a cytoplasmic marker, and Histone H3 served as a nuclear marker to verify enrichment success. Protein samples from three biological replicates per dose were analyzed.

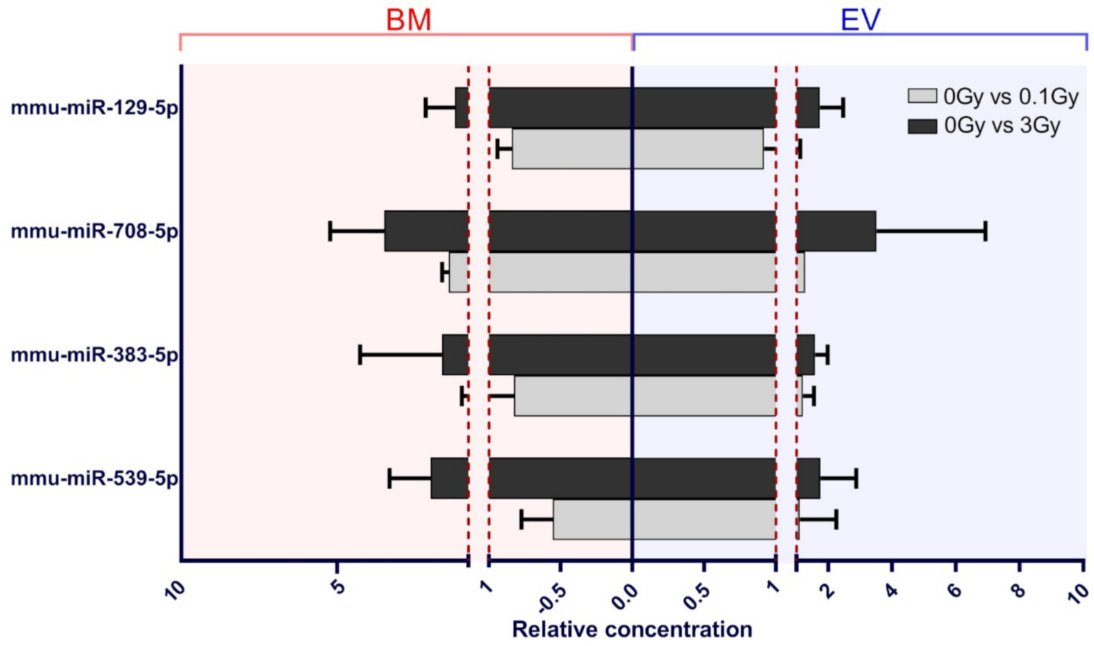

Supplementary Figure 7: Non-signficant BM and BM-dervied EV miRNAs. miRNAs without significant concentration alterations after 0.1Gy or 3Gy IR compared to 0Gy control groups.

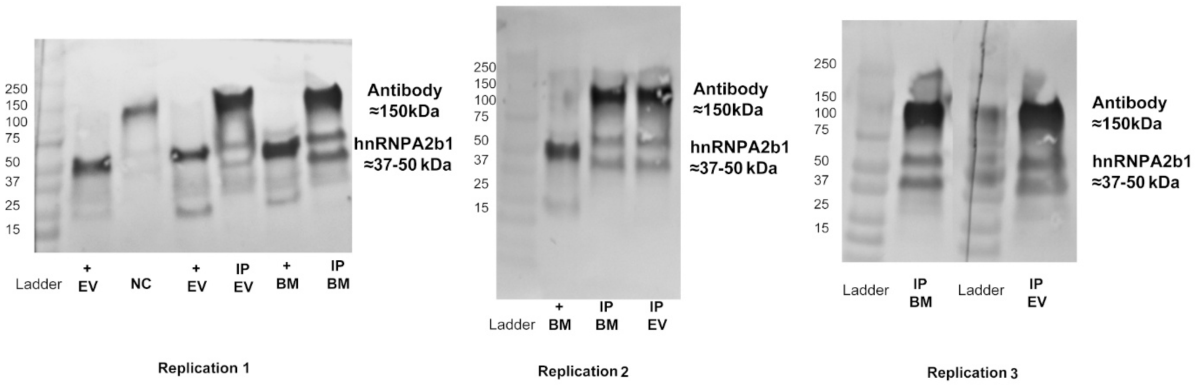

Supplementary Figure 8: Western blot detection of immunoprecipitated hnRNP A2B1 protein. NC represents the negative control, where IP was performed using PBS instead of BM or BM-EV samples, confirming that the hnRNP A2B1 antibody signal does not interfere with the protein signal. **+0 Gy EV** and **+0 Gy BM** indicate the presence of hnRNP A2B1 in non-immunoprecipitated samples, while **IP 0 Gy EV** and **IP 0 Gy BM** demonstrate the presence of hnRNP A2B1 after immunoprecipitation
