# Supplementary material for: Ionizing Radiation Actively Reshapes Bone Marrow-Derived Extracellular Vesicle MicroRNA Cargo with the Involvement of hnRNP A2b1
Source: Int J Mol Sci. 2026 Jun 18;27(12):5510. doi: 10.3390/ijms27125510 (PMC13299799; doi:10.3390/ijms27125510)
Supplement: Supplementary file 1 [file ijms-27-05510-s001.zip › Supplementary Table 1m.pdf]

Supplementary Table 1: RNA-binding proteins and their motifs associated with miRNA sorting and packaging into EVs

RNA-binding protein	Identified motif /interacting partner	Source
hnRNPA2B1	GGAG, AGGUAG	13,19, 37, 42
hnRNPQ	GGCU/A	11,27,42
hnRNPH1	5′-GGGA	35,42
hnRNPC1	AU-rich elements (AREs)	32,42
hnRNPK	5′-UC3–4(U/A)2	33,42
ANXA2	5'-AA(C/G)(A/U)G	14,21,42
AGO2	G-rich sequences / 5′-GCACUU or without motif	25,42,44
HuR	AU-rich elements (AREs)	41,42
NCL	5'-UUAGGG	39,42
TDP-43	9 "UG" rep (*highest affinity),* 3 "UG" rep *(lower affinity),* 2 "UG" rep *(weak affinity)* GU rich sequence (GUGU)	28,40,42
LIN28	5'-GGAGG 5'-GGAGA	36,42
YBX1	5′-ACCAGCCU, 5′-CAGUGAGC, 5′-UAAUCCCA	29,42,45
FUS	GGUG, CGCGC, GUGGU	26, 30,,34,42
ALIX	*interaction without specific motif*	42,43
MVP	*interaction without specific motif*	38,42
