# Supplementary material for: Ionizing Radiation Actively Reshapes Bone Marrow-Derived Extracellular Vesicle MicroRNA Cargo with the Involvement of hnRNP A2b1
Source: Int J Mol Sci. 2026 Jun 18;27(12):5510. doi: 10.3390/ijms27125510 (PMC13299799; doi:10.3390/ijms27125510)
Supplement: Supplementary file 1 [file ijms-27-05510-s001.zip › Supplementary Table 2.pdf]

Supplementary Table 2A: **KEGG pathways analysis of significantly deregulated EV-miRNAs 24h after 3Gy IR.**

Pathway	Category	Subgroup	Target Genes (n)	Genes/ Pathway (n)	Norm. Coverage	miRNAs (n)	Norm. miRNA	Gene ratio	FDR p-value	Score	Rank
MicroRNAs in cancer	Human Diseases	Cancers: Overview	139	164	0,8377	16	1	0,8377	1E-17	0,9459	1
Prostate cancer	Human Diseases	Cancers: Specific types	86	103	0,80	14	0,83	0,96	2E-10	0,72	2
Renal cell carcinoma	Human Diseases	Cancers: Specific types	61	69	0,95	11	0,58	1,63	1E-09	0,67	3
Pancreatic cancer	Human Diseases	Cancers: Specific types	66	78	0,83	13	0,75	1,11	8E-09	0,67	4
Chronic myeloid leukemia	Human Diseases	Cancers: Specific types	65	77	0,83	13	0,75	1,10	1E-08	0,67	5
Pathways in cancer	Human Diseases	Cancers: Overview	371	555	0,28	16	1,00	0,28	3E-13	0,67	6
Apoptosis	Cellular Processes	Cell growth and death	113	137	0,77	9	0,42	1,84	7E-13	0,62	7
Viral carcinogenesis	Human Diseases	Cancers: Overview	163	226	0,45	15	0,92	0,49	1E-09	0,62	8
Glioma	Human Diseases	Cancers: Specific types	62	76	0,74	13	0,75	0,99	2E-07	0,61	9
IL-17 signaling pathway	Environmental Information Processing	Signal transduction	74	94	0,65	14	0,83	0,78	2E-07	0,61	10
MAPK signaling pathway	Environmental Information Processing	Signal transduction	209	302	0,36	15	0,92	0,39	1E-09	0,59	11
Colorectal cancer	Human Diseases	Cancers: Specific types	73	90	0,72	11	0,58	1,24	3E-08	0,57	12
Non-small cell lung cancer	Human Diseases	Cancers: Specific types	61	74	0,77	11	0,58	1,31	1E-07	0,57	13
FoxO signaling pathway	Environmental Information Processing	Signal transduction	106	136	0,63	11	0,58	1,07	1E-09	0,56	14
Endocrine resistance	Human Diseases	Cancers: Overview	75	95	0,66	12	0,67	0,99	1E-07	0,56	15
Cellular senescence	Cellular Processes	Cell growth and death	131	182	0,44	14	0,83	0,53	5E-08	0,55	16
Cell cycle	Cellular Processes	Cell growth and death	94	125	0,54	13	0,75	0,72	2E-07	0,55	17
HIF-1 signaling pathway	Environmental Information Processing	Signal transduction	89	116	0,59	12	0,67	0,88	9E-08	0,54	18
EGFR tyrosine kinase inhibitor resistance	Human Diseases	Cancers: Overview	67	82	0,74	10	0,50	1,49	7E-08	0,54	19
Human T-cell leukemia virus 1 infection	Human Diseases	Infectious disease: Viral	167	244	0,33	15	0,92	0,36	1E-07	0,53	20
Th17 cell differentiation	Organismal Systems	Immune system	76	105	0,45	15	0,92	0,50	2E-05	0,53	21
Endocytosis	Cellular Processes	Transport and catabolism	186	267	0,37	13	0,75	0,49	4E-09	0,52	22

PD-L1 expression and PD-1 checkpoint pathway in cancer	Human Diseases	Cancers: Overview	74	91	0,73	9	0,42	1,75	2E-08	0,52	23
Fluid shear stress and atherosclerosis	Human Diseases	Cardiovascular disease	114	149	0,58	10	0,50	1,16	2E-09	0,52	24
Platinum drug resistance	Human Diseases	Cancers: Overview	65	84	0,61	12	0,67	0,91	3E-06	0,51	25
Autophagy - animal	Cellular Processes	Transport and catabolism	112	144	0,62	9	0,42	1,49	8E-10	0,51	26
Rap1 signaling pathway	Environmental Information Processing	Signal transduction	150	217	0,35	14	0,83	0,42	2E-07	0,51	27
Hepatitis B	Human Diseases	Infectious disease: Viral	119	166	0,43	13	0,75	0,58	2E-07	0,51	28
Proteoglycans in cancer	Human Diseases	Cancers: Overview	148	207	0,43	12	0,67	0,64	1E-08	0,50	29
Axon guidance	Organismal Systems	Development and regeneration	129	182	0,41	13	0,75	0,54	2E-07	0,50	30
Focal adhesion	Cellular Processes	Cell motility	151	205	0,49	10	0,50	0,99	6E-10	0,50	31
T cell receptor signaling pathway	Organismal Systems	Immune system	84	106	0,67	9	0,42	1,60	2E-08	0,50	32
Salmonella infection	Human Diseases	Infectious disease: Bacterial	182	253	0,44	10	0,50	0,88	2E-10	0,49	33
PI3K-Akt signaling pathway	Environmental Information Processing	Signal transduction	239	364	0,25	14	0,83	0,30	5E-08	0,49	34
Human cytomegalovirus infection	Human Diseases	Infectious disease: Viral	170	253	0,29	14	0,83	0,35	5E-07	0,48	35
Ubiquitin mediated proteolysis	Genetic Information Processing	Folding, sorting and degradation	101	145	0,37	14	0,83	0,44	1E-05	0,48	36
Neurotrophin signaling pathway	Organismal Systems	Nervous system	90	123	0,48	12	0,67	0,72	2E-06	0,47	37
Toxoplasmosis	Human Diseases	Infectious disease: Parasitic	84	112	0,54	11	0,58	0,92	9E-07	0,47	38
p53 signaling pathway	Cellular Processes	Cell growth and death	59	72	0,75	8	0,33	2,25	3E-07	0,47	39
Kaposi sarcoma-associated herpesvirus	Human Diseases	Infectious disease: Viral	145	219	0,26	15	0,92	0,29	1E-05	0,47	40
Adherens junction	Cellular Processes	Cell motility	56	71	0,66	10	0,50	1,31	6E-06	0,47	41
Endometrial cancer	Human Diseases	Cancers: Specific types	48	59	0,73	9	0,42	1,76	6E-06	0,47	42
Chagas disease	Human Diseases	Infectious disease: Parasitic	78	104	0,54	11	0,58	0,92	2E-06	0,46	43
Protein processing in endoplasmic reticulum	Genetic Information Processing	Folding, sorting and degradation	124	172	0,45	11	0,58	0,76	9E-08	0,46	44

TNF signaling pathway	Environmental Information Processing	Signal transduction	93	119	0,63	8	0,33	1,90	1E-08	0,46	45
Ras signaling pathway	Environmental Information Processing	Signal transduction	154	235	0,24	15	0,92	0,26	1E-05	0,46	46
Insulin resistance	Human Diseases	Endocrine and metabolic disease	82	115	0,42	13	0,75	0,56	3E-05	0,46	47
Hepatocellular carcinoma	Human Diseases	Cancers: Specific types	125	181	0,35	13	0,75	0,47	3E-06	0,46	48
Steroid biosynthesis	Metabolism	Lipid metabolism	18	20	1,00	7	0,25	4,00	0,001	0,45	49
Melanoma	Human Diseases	Cancers: Specific types	54	73	0,50	12	0,67	0,76	0,0002	0,44	50
Ferroptosis	Cellular Processes	Cell growth and death	32	40	0,69	10	0,50	1,38	0,0005	0,44	51
Yersinia infection	Human Diseases	Infectious disease: Bacterial	98	136	0,44	11	0,58	0,76	2E-06	0,44	52
Thyroid hormone signaling pathway	Organismal Systems	Endocrine system	90	122	0,50	10	0,50	1,00	1E-06	0,43	53
ErbB signaling pathway	Environmental Information Processing	Signal transduction	67	87	0,60	9	0,42	1,43	3E-06	0,43	54
Acute myeloid leukemia	Human Diseases	Cancers: Specific types	56	72	0,62	9	0,42	1,49	1E-05	0,42	55
AGE-RAGE signaling pathway in diabetic complications	Human Diseases	Endocrine and metabolic disease	75	103	0,47	11	0,58	0,80	2E-05	0,42	56
Th1 and Th2 cell differentiation	Organismal Systems	Immune system	68	89	0,58	9	0,42	1,39	4E-06	0,42	57
Bladder cancer	Human Diseases	Cancers: Specific types	32	41	0,63	10	0,50	1,26	0,0011	0,41	58
Choline metabolism in cancer	Human Diseases	Cancers: Overview	78	102	0,58	8	0,33	1,74	7E-07	0,41	59
Small cell lung cancer	Human Diseases	Cancers: Specific types	73	97	0,54	9	0,42	1,30	4E-06	0,41	60
Fatty acid metabolism	Metabolism	Lipid metabolism	49	62	0,66	8	0,33	1,98	2E-05	0,40	61
AMPK signaling pathway	Environmental Information Processing	Signal transduction	95	130	0,48	9	0,42	1,14	1E-06	0,40	62
mTOR signaling pathway	Environmental Information Processing	Signal transduction	108	163	0,26	13	0,75	0,35	0,0002	0,39	63
Human immunodeficiency virus 1 infection	Human Diseases	Infectious disease: Viral	157	238	0,26	12	0,67	0,38	7E-06	0,39	64
Adipocytokine signaling pathway	Organismal Systems	Endocrine system	55	71	0,61	8	0,33	1,84	2E-05	0,39	65
VEGF signaling pathway	Environmental Information Processing	Signal transduction	47	60	0,64	8	0,33	1,92	5E-05	0,39	66
Apoptosis - multiple species	Cellular Processes	Cell growth and death	26	32	0,73	8	0,33	2,19	0,0013	0,39	67

Fc epsilon RI signaling pathway	Organismal Systems	Immune system	52	67	0,62	8	0,33	1,85	3E-05	0,39	68
Progesterone-mediated oocyte maturation	Organismal Systems	Endocrine system	60	94	0,19	15	0,92	0,21	0,0165	0,38	69
Lysosome	Cellular Processes	Transport and catabolism	95	132	0,44	9	0,42	1,06	3E-06	0,38	70
Gastric cancer	Human Diseases	Cancers: Specific types	99	151	0,24	13	0,75	0,32	0,0006	0,37	71
RNA degradation	Genetic Information Processing	Transcription	54	83	0,23	14	0,83	0,27	0,0139	0,37	72
Breast cancer	Human Diseases	Cancers: Specific types	100	150	0,28	12	0,67	0,42	0,0002	0,36	73
TGF-beta signaling pathway	Environmental Information Processing	Signal transduction	67	96	0,37	11	0,58	0,64	0,0005	0,36	74
Regulation of actin cytoskeleton	Cellular Processes	Cell motility	147	222	0,26	11	0,58	0,45	1E-05	0,36	75
Growth hormone synthesis, secretion and action	Organismal Systems	Endocrine system	83	118	0,39	10	0,50	0,78	5E-05	0,36	76
Central carbon metabolism in cancer	Human Diseases	Cancers: Overview	53	70	0,56	8	0,33	1,67	7E-05	0,36	77
Lysine degradation	Metabolism	Amino acid metabolism	48	63	0,57	8	0,33	1,72	0,0001	0,36	78
Insulin signaling pathway	Organismal Systems	Endocrine system	97	143	0,31	11	0,58	0,54	0,0001	0,36	79
Relaxin signaling pathway	Organismal Systems	Endocrine system	87	131	0,27	12	0,67	0,40	0,0007	0,35	80
Phosphatidylinositol signaling system	Environmental Information Processing	Signal transduction	72	100	0,44	9	0,42	1,06	5E-05	0,35	81
Longevity regulating pathway	Organismal Systems	Aging	68	92	0,50	8	0,33	1,51	2E-05	0,35	82
DNA replication	Genetic Information Processing	Replication and repair	28	36	0,62	8	0,33	1,86	0,0026	0,35	83
Hippo signaling pathway - multiple species	Environmental Information Processing	Signal transduction	22	28	0,65	8	0,33	1,94	0,0067	0,34	84
Oocyte meiosis	Organismal Systems	Development and regeneration	75	123	0,10	15	0,92	0,11	0,03	0,34	85
Human papillomavirus infection	Human Diseases	Infectious disease: Viral	229	361	0,18	11	0,58	0,30	3E-06	0,34	86
C-type lectin receptor signaling pathway	Organismal Systems	Immune system	79	114	0,36	10	0,50	0,72	0,0002	0,34	87
Metabolic pathways	Metabolism	Global and overview maps	918	1591	0,00	11	0,58	0,00	1E-08	0,34	88

Base excision repair	Genetic Information Processing	Replication and repair	27	35	0,60	8	0,33	1,81	0,0039	0,34	89
Terpenoid backbone biosynthesis	Metabolism	Metabolism of terpenoids and polyketides	18	23	0,64	8	0,33	1,91	0,0154	0,33	90
Pathways of neurodegeneration - multiple diseases	Human Diseases	Neurodegenerative disease	288	480	0,07	13	0,75	0,09	9E-05	0,33	91
Inositol phosphate metabolism	Metabolism	Carbohydrate metabolism	53	72	0,49	8	0,33	1,48	0,0002	0,32	92
Longevity regulating pathway - multiple species	Organismal Systems	Aging	47	64	0,49	8	0,33	1,46	0,0007	0,31	93
Herpes simplex virus 1 infection	Human Diseases	Infectious disease: Viral	287	453	0,18	9	0,42	0,42	2E-07	0,31	94
Circadian rhythm	Organismal Systems	Circadian rhythm	24	30	0,69	6	0,17	4,14	0,003	0,31	95
Epstein-Barr virus infection	Human Diseases	Infectious disease: Viral	145	227	0,19	11	0,58	0,33	0,0002	0,31	96
JAK-STAT signaling pathway	Environmental Information Processing	Signal transduction	105	170	0,13	13	0,75	0,17	0,0067	0,31	97
Fc gamma R-mediated phagocytosis	Organismal Systems	Immune system	68	95	0,43	8	0,33	1,29	0,0001	0,31	98
Osteoclast differentiation	Organismal Systems	Development and regeneration	84	128	0,25	11	0,58	0,42	0,0015	0,31	99
Signaling pathways regulating pluripotency of stem cells	Organismal Systems	Development and regeneration	88	142	0,13	13	0,75	0,18	0,0109	0,31	100
Leishmaniasis	Human Diseases	Infectious disease: Parasitic	47	70	0,29	11	0,58	0,50	0,0099	0,31	101
Glyoxylate and dicarboxylate metabolism	Metabolism	Carbohydrate metabolism	24	31	0,61	7	0,25	2,44	0,0062	0,31	102
Thermogenesis	Organismal Systems	Circadian rhythm	141	234	0,08	13	0,75	0,11	0,0057	0,30	103
Vasopressin-regulated water reabsorption	Organismal Systems	Excretory system	34	44	0,61	6	0,17	3,64	0,001	0,29	104
Coronavirus disease - COVID-19	Human Diseases	Infectious disease: Viral	151	246	0,11	12	0,67	0,17	0,0015	0,29	105
Thyroid cancer	Human Diseases	Cancers: Specific types	28	37	0,56	7	0,25	2,23	0,005	0,29	106
Sphingolipid signaling pathway	Environmental Information Processing	Signal transduction	85	129	0,25	10	0,50	0,51	0,0012	0,29	107
Mitophagy - animal	Cellular Processes	Transport and catabolism	47	66	0,42	8	0,33	1,26	0,0019	0,28	108
Hepatitis C	Human Diseases	Infectious disease: Viral	101	164	0,12	12	0,67	0,18	0,0082	0,28	109

Pertussis	Human Diseases	Infectious disease: Bacterial	52	76	0,33	9	0,42	0,80	0,0041	0,27	110
Prolactin signaling pathway	Organismal Systems	Endocrine system	52	76	0,33	9	0,42	0,80	0,0041	0,27	111
Long-term depression	Organismal Systems	Nervous system	42	61	0,35	9	0,42	0,83	0,0082	0,27	112
Tuberculosis	Human Diseases	Infectious disease: Bacterial	110	181	0,10	12	0,67	0,14	0,0099	0,27	113
Measles	Human Diseases	Infectious disease: Viral	99	147	0,30	8	0,33	0,90	0,0001	0,27	114
Porphyrin and chlorophyll metabolism	Metabolism	Metabolism of other amino acids	30	41	0,48	7	0,25	1,92	0,0077	0,26	115
Gap junction	Cellular Processes	Cell motility	60	87	0,35	8	0,33	1,05	0,0015	0,26	116
Hippo signaling pathway	Environmental Information Processing	Signal transduction	102	160	0,19	10	0,50	0,37	0,0019	0,26	117
GnRH signaling pathway	Organismal Systems	Endocrine system	61	91	0,29	9	0,42	0,69	0,0038	0,26	118
Autophagy - other	Cellular Processes	Transport and catabolism	25	34	0,49	7	0,25	1,96	0,0139	0,26	119
Estrogen signaling pathway	Organismal Systems	Endocrine system	84	135	0,14	11	0,58	0,24	0,0111	0,25	120
NF-kappa B signaling pathway	Environmental Information Processing	Signal transduction	72	106	0,32	8	0,33	0,95	0,0009	0,25	121
Toll-like receptor signaling pathway	Organismal Systems	Immune system	68	100	0,32	8	0,33	0,96	0,0012	0,25	122
Valine, leucine and isoleucine degradation	Metabolism	Amino acid metabolism	39	56	0,37	8	0,33	1,11	0,0082	0,25	123
Non-alcoholic fatty liver disease	Human Diseases	Endocrine and metabolic disease	97	153	0,18	10	0,50	0,35	0,0032	0,25	124
Propanoate metabolism	Metabolism	Carbohydrate metabolism	24	34	0,40	8	0,33	1,20	0,0321	0,25	125
Glucagon signaling pathway	Organismal Systems	Endocrine system	67	105	0,19	10	0,50	0,38	0,0111	0,24	126
Cholesterol metabolism	Metabolism	Lipid metabolism	33	49	0,30	9	0,42	0,72	0,0299	0,24	127
B cell receptor signaling pathway	Organismal Systems	Immune system	53	78	0,32	8	0,33	0,95	0,0047	0,24	128
Alzheimer disease	Human Diseases	Neurodegenerative disease	217	376	0,00	12	0,67	0,00	0,0088	0,24	129
Glycerolipid metabolism	Metabolism	Lipid metabolism	43	63	0,33	8	0,33	0,98	0,0092	0,24	130
Cushing syndrome	Human Diseases	Endocrine and metabolic disease	100	165	0,09	11	0,58	0,15	0,0155	0,24	131
Parathyroid hormone synthesis, secretion and action	Organismal Systems	Endocrine system	74	111	0,28	8	0,33	0,83	0,0016	0,24	132

Amino sugar and nucleotide sugar metabolism	Metabolism	Carbohydrate metabolism	36	51	0,40	7	0,25	1,60	0,0082	0,23	133
Glycosphingolipid biosynthesis - ganglio series	Metabolism	Lipid metabolism	12	15	0,69	4	0,00	3,60	0,0401	0,23	134
Notch signaling pathway	Environmental Information Processing	Signal transduction	38	54	0,39	7	0,25	1,57	0,0073	0,23	135
Glutathione metabolism	Metabolism	Metabolism of other amino acids	50	74	0,31	8	0,33	0,92	0,0069	0,23	136
Alanine, aspartate and glutamate metabolism	Metabolism	Amino acid metabolism	27	38	0,41	7	0,25	1,65	0,0203	0,23	137
Nucleotide excision repair	Genetic Information Processing	Replication and repair	32	44	0,47	6	0,17	2,79	0,0069	0,23	138
Prion disease	Human Diseases	Neurodegenerative disease	161	272	0,05	11	0,58	0,08	0,0077	0,23	139
Alcoholism	Human Diseases	Substance dependence	119	201	0,05	11	0,58	0,08	0,0212	0,22	140
Adrenergic signaling in cardiomyocytes	Organismal Systems	Cardiovascular system	94	158	0,06	11	0,58	0,10	0,0338	0,22	141
Amyotrophic lateral sclerosis	Human Diseases	Neurodegenerative disease	217	374	0,01	11	0,58	0,02	0,0068	0,22	142
Aldosterone-regulated sodium reabsorption	Organismal Systems	Excretory system	27	40	0,30	8	0,33	0,91	0,0465	0,21	143
Chemokine signaling pathway	Environmental Information Processing	Signal transduction	120	193	0,14	9	0,42	0,33	0,0027	0,21	144
Other glycan degradation	Metabolism	Glycan biosynthesis and metabolism	14	18	0,62	4	0,00	3,71	0,0364	0,21	145
Peroxisome	Cellular Processes	Transport and catabolism	59	90	0,24	8	0,33	0,73	0,0082	0,21	146
Biosynthesis of cofactors	Metabolism	Metabolism of vitamins and cofactors	100	156	0,20	8	0,33	0,59	0,0016	0,21	147
Tryptophan metabolism	Metabolism	Amino acid metabolism	35	51	0,34	7	0,25	1,35	0,017	0,21	148
Glycerophospholipid metabolism	Metabolism	Lipid metabolism	65	100	0,23	8	0,33	0,68	0,0074	0,20	149
Glycosylphosphatidylinositol (GPI)-anchor biosynthesis	Metabolism	Glycan biosynthesis and metabolism	18	25	0,44	6	0,17	2,66	0,0488	0,20	150
Fatty acid degradation	Metabolism	Lipid metabolism	35	53	0,26	8	0,33	0,77	0,036	0,20	151
Carbon metabolism	Metabolism	Global and overview maps	79	123	0,20	8	0,33	0,61	0,005	0,20	152
Platelet activation	Organismal Systems	Cardiovascular system	82	128	0,20	8	0,33	0,59	0,0047	0,20	153

Hypertrophic cardiomyopathy	Human Diseases	Cardiovascular disease	59	94	0,16	9	0,42	0,38	0,0271	0,20	154
Amoebiasis	Human Diseases	Infectious disease: Parasitic	66	109	0,09	10	0,50	0,18	0,0474	0,20	155
Dopaminergic synapse	Organismal Systems	Nervous system	84	140	0,07	10	0,50	0,14	0,0347	0,19	156
Amphetamine addiction	Human Diseases	Substance dependence	46	71	0,22	8	0,33	0,66	0,0258	0,19	157
Huntington disease	Human Diseases	Neurodegenerative disease	180	308	0,02	10	0,50	0,05	0,0092	0,19	158
Type II diabetes mellitus	Human Diseases	Endocrine and metabolic disease	33	49	0,30	7	0,25	1,19	0,0299	0,19	159
Phospholipase D signaling pathway	Environmental Information Processing	Signal transduction	95	151	0,16	8	0,33	0,48	0,0049	0,19	160
Viral myocarditis	Human Diseases	Cardiovascular disease	51	82	0,14	9	0,42	0,33	0,0465	0,19	161
Apelin signaling pathway	Environmental Information Processing	Signal transduction	84	137	0,11	9	0,42	0,27	0,0184	0,19	162
cGMP-PKG signaling pathway	Environmental Information Processing	Signal transduction	103	175	0,04	10	0,50	0,07	0,0368	0,18	163
Legionellosis	Human Diseases	Infectious disease: Bacterial	41	60	0,33	6	0,17	1,98	0,0107	0,18	164
Transcriptional misregulation in cancer	Human Diseases	Cancers: Overview	125	214	0,02	10	0,50	0,04	0,03	0,18	165
Bacterial invasion of epithelial cells	Human Diseases	Infectious disease: Bacterial	49	77	0,18	8	0,33	0,55	0,0321	0,18	166
Leukocyte transendothelial migration	Organismal Systems	Immune system	73	118	0,13	8	0,33	0,39	0,0216	0,16	167
Oxytocin signaling pathway	Organismal Systems	Endocrine system	94	158	0,06	9	0,42	0,13	0,0338	0,16	168
Melanogenesis	Organismal Systems	Endocrine system	63	103	0,11	8	0,33	0,32	0,0418	0,15	169
Biosynthesis of amino acids	Metabolism	Global and overview maps	50	79	0,17	7	0,25	0,69	0,0341	0,14	170
Neutrophil extracellular trap formation	Organismal Systems	Immune system	120	203	0,04	8	0,33	0,13	0,0217	0,13	171
Fanconi anemia pathway	Genetic Information Processing	Replication and repair	35	52	0,30	5	0,08	3,57	0,0257	0,13	172
N-Glycan biosynthesis	Metabolism	Glycan biosynthesis and metabolism	35	52	0,30	5	0,08	3,57	0,0257	0,13	173
Various types of N-glycan biosynthesis	Metabolism	Glycan biosynthesis and metabolism	27	40	0,30	5	0,08	3,64	0,0465	0,13	174
Galactose metabolism	Metabolism	Carbohydrate metabolism	23	33	0,37	4	0,00	3,71	0,0422	0,13	175
Fructose and mannose metabolism	Metabolism	Carbohydrate metabolism	25	36	0,36	4	0,00	3,71	0,0364	0,12	176

SNARE interactions in vesicular transport	Cellular Processes	Transport and catabolism	24	35	0,34	4	0,00	3,71	0,048	0,11	177
Spinocerebellar ataxia	Human Diseases	Neurodegenerative disease	88	148	0,05	7	0,25	0,22	0,0399	0,10	178

Supplementary Table 2B: **KEGG pathways analysis of significantly deregulated hnRNP A2b1 related miRNAs**.

Pathway	Category	Subgroup	Target Genes (n)	Genes/ Pathway (n)	Norm. Coverage	miRNAs (n)	Norm. miRNA	Gene ratio	FDR p-value	Score	Rank
**MicroRNAs in cancer**	Human Diseases	Cancers: Overview	84	164	0,51	12	0,86	0,59	2,51 E-14	0,79	1
**Pathways in cancer**	Human Diseases	Cancers: Overview	181	555	0,32	13	1,00	0,32	2,75 E-08	0,77	2
**Renal cell carcinoma**	Human Diseases	Cancers: Specific types	41	69	0,59	11	0,71	0,82	1,14 E-09	0,77	3
**Focal adhesion**	Cellular Processes	Cellular community - eukaryotes	84	205	0,40	12	0,86	0,47	1,48 E-08	0,75	4
**MAPK signaling pathway**	Environmental Information Processing	Signal transduction	116	302	0,38	12	0,86	0,44	1,53 E-09	0,74	5
**Chronic myeloid leukemia**	Human Diseases	Cancers: Specific types	40	77	0,51	11	0,71	0,72	1,42 E-07	0,74	6
**Signaling pathways regulating pluripotency of stem cells**	Organismal Systems	Development and regeneration	51	142	0,35	12	0,86	0,41	4,09 E-04	0,73	7
**Salmonella infection**	Human Diseases	Infectious disease: Bacterial	88	253	0,34	12	0,86	0,40	9,41 E-06	0,73	8
**Acute myeloid leukemia**	Human Diseases	Cancers: Specific types	34	72	0,47	11	0,71	0,65	1,20 E-05	0,73	9
**ErbB signaling pathway**	Environmental Information Processing	Signal transduction	41	87	0,46	11	0,71	0,65	2,08 E-06	0,73	10
**T cell receptor signaling pathway**	Organismal Systems	Immune system	49	106	0,46	11	0,71	0,64	3,41 E-07	0,72	11
**Prolactin signaling pathway**	Organismal Systems	Endocrine system	35	76	0,45	11	0,71	0,64	1,63 E-05	0,72	12
**Thyroid cancer**	Human Diseases	Cancers: Specific types	18	37	0,48	11	0,71	0,67	1,21 E-03	0,72	13
**Autophagy - animal**	Cellular Processes	Transport and catabolism	62	144	0,42	11	0,71	0,59	1,62 E-07	0,71	14
**Insulin resistance**	Human Diseases	Endocrine and metabolic disease	49	115	0,42	11	0,71	0,59	4,40 E-06	0,71	15
**AMPK signaling pathway**	Environmental Information Processing	Signal transduction	54	130	0,41	11	0,71	0,57	3,57 E-06	0,71	16
**Neurotrophin signaling pathway**	Organismal Systems	Nervous system	51	123	0,41	11	0,71	0,57	6,30 E-06	0,71	17
**D-Glutamine and D-glutamate metabolism**	Metabolism	Amino acid metabolism	4	40	0,09	8	0,29	0,31	7,50 E-03	0,40	18
**Yersinia infection**	Human Diseases	Infectious disease: Bacterial	55	136	0,40	11	0,71	0,56	6,30 E-06	0,70	19

**Alzheimer disease**	Human Diseases	Neurodegenerative disease	110	376	0,28	12	0,86	0,33	1,45 E-03	0,70	20
**JAK-STAT signaling pathway**	Environmental Information Processing	Signal transduction	55	170	0,31	12	0,86	0,37	3,23 E-03	0,70	21
**Prostate cancer**	Human Diseases	Cancers: Specific types	55	103	0,53	10	0,57	0,92	2,18 E-10	0,70	22
**TGF-beta signaling pathway**	Environmental Information Processing	Signal transduction	38	96	0,39	11	0,71	0,54	3,25 E-04	0,70	23
**Proteoglycans in cancer**	Human Diseases	Cancers: Overview	77	207	0,36	11	0,71	0,51	3,41 E-06	0,69	24
**Pancreatic cancer**	Human Diseases	Cancers: Specific types	40	78	0,51	10	0,57	0,89	1,90 E-07	0,69	25
**PD-L1 expression and PD-1 checkpoint pathway in cancer**	Human Diseases	Cancers: Overview	46	91	0,50	10	0,57	0,87	3,57 E-08	0,69	26
**Endocrine resistance**	Human Diseases	Drug resistance: Antineoplastic	48	95	0,50	10	0,57	0,87	2,31 E-08	0,69	27
**Spinocerebellar ataxia**	Human Diseases	Neurodegenerative disease	54	148	0,36	11	0,71	0,50	1,76 E-04	0,69	28
**Endometrial cancer**	Human Diseases	Cancers: Specific types	29	59	0,49	10	0,57	0,85	2,32 E-05	0,69	29
**Human cytomegalovirus infection**	Human Diseases	Infectious disease: Viral	88	253	0,34	11	0,71	0,48	9,41 E-06	0,68	30
**Non-alcoholic fatty liver disease**	Human Diseases	Endocrine and metabolic disease	54	153	0,34	11	0,71	0,48	4,25 E-04	0,68	31
**Endocytosis**	Cellular Processes	Transport and catabolism	91	267	0,33	11	0,71	0,47	1,56 E-05	0,68	32
**Phospholipase D signaling pathway**	Environmental Information Processing	Signal transduction	53	151	0,34	11	0,71	0,48	5,63 E-04	0,68	33
**Adipocytokine signaling pathway**	Organismal Systems	Endocrine system	34	71	0,47	10	0,57	0,83	8,58 E-06	0,68	34
**Colorectal cancer**	Human Diseases	Cancers: Specific types	43	90	0,47	10	0,57	0,82	7,24 E-07	0,68	35
**EGFR tyrosine kinase inhibitor resistance**	Human Diseases	Drug resistance: Antineoplastic	39	82	0,47	10	0,57	0,82	2,72 E-06	0,68	36
**Hepatocellular carcinoma**	Human Diseases	Cancers: Specific types	62	181	0,33	11	0,71	0,47	3,79 E-04	0,68	37
**Glioma**	Human Diseases	Cancers: Specific types	36	76	0,47	10	0,57	0,82	6,30 E-06	0,68	38
**FoxO signaling pathway**	Environmental Information Processing	Signal transduction	63	136	0,46	10	0,57	0,80	7,32 E-09	0,68	39
**Non-small cell lung cancer**	Human Diseases	Cancers: Specific types	34	74	0,45	10	0,57	0,79	2,32 E-05	0,67	40
**AGE-RAGE signaling pathway in diabetic complications**	Human Diseases	Endocrine and metabolic disease	47	103	0,45	10	0,57	0,79	9,84 E-07	0,67	41
**mTOR signaling pathway**	Environmental Information Processing	Signal transduction	55	163	0,33	11	0,71	0,46	1,21 E-03	0,67	42
**Thyroid hormone signaling pathway**	Organismal Systems	Endocrine system	55	122	0,44	10	0,57	0,78	1,62 E-07	0,67	43
**Regulation of actin cytoskeleton**	Cellular Processes	Cell motility	72	222	0,32	11	0,71	0,44	6,79 E-04	0,67	44
**Longevity regulating pathway**	Organismal Systems	Aging	41	92	0,44	10	0,57	0,77	8,04 E-06	0,67	45
**Small cell lung cancer**	Human Diseases	Cancers: Specific types	42	97	0,43	10	0,57	0,75	1,30 E-05	0,67	46
**TNF signaling pathway**	Environmental	Signal transduction	51	119	0,42	10	0,57	0,74	2,72 E-06	0,66	47

	Information Processing										
**Hepatitis B**	Human Diseases	Infectious disease: Viral	71	166	0,42	10	0,57	0,74	2,75 E-08	0,66	48
**Growth hormone synthesis, secretion and action**	Organismal Systems	Endocrine system	50	118	0,42	10	0,57	0,73	4,26 E-06	0,66	49
**Human immunodeficiency virus 1 infection**	Human Diseases	Infectious disease: Viral	74	238	0,30	11	0,71	0,42	1,90 E-03	0,66	50
**Epstein-Barr virus infection**	Human Diseases	Infectious disease: Viral	71	227	0,30	11	0,71	0,43	1,99 E-03	0,66	51
**Ubiquitin mediated proteolysis**	Genetic Information Processing	Folding, sorting and degradation	59	145	0,40	10	0,57	0,70	2,72 E-06	0,66	52
**Ras signaling pathway**	Environmental Information Processing	Signal transduction	73	235	0,30	11	0,71	0,42	2,05 E-03	0,66	53
**Insulin signaling pathway**	Organismal Systems	Endocrine system	58	143	0,40	10	0,57	0,70	3,57 E-06	0,66	54
**Human T-cell leukemia virus 1 infection**	Human Diseases	Infectious disease: Viral	97	244	0,39	10	0,57	0,68	6,37 E-09	0,65	55
**Pathways of neurodegeneration - multiple diseases**	Human Diseases	Neurodegenerative disease	136	480	0,27	11	0,71	0,38	1,45 E-03	0,65	56
**Chagas disease**	Human Diseases	Infectious disease: Parasitic	41	104	0,39	10	0,57	0,68	2,01 E-04	0,65	57
**Osteoclast differentiation**	Organismal Systems	Development and regeneration	50	128	0,38	10	0,57	0,67	4,57 E-05	0,65	58
**Glucagon signaling pathway**	Organismal Systems	Endocrine system	41	105	0,38	10	0,57	0,67	2,53 E-04	0,65	59
**Axon guidance**	Organismal Systems	Nervous system	69	182	0,37	10	0,57	0,65	4,94 E-06	0,65	60
**Mitophagy - animal**	Cellular Processes	Transport and catabolism	27	66	0,40	10	0,57	0,70	1,47 E-03	0,65	61
**Circadian rhythm**	Organismal Systems	Environmental adaptation	16	30	0,53	9	0,43	1,23	7,26 E-04	0,65	62
**Choline metabolism in cancer**	Human Diseases	Cancers: Overview	39	102	0,37	10	0,57	0,66	5,64 E-04	0,64	63
**Relaxin signaling pathway**	Organismal Systems	Endocrine system	48	131	0,36	10	0,57	0,63	3,79 E-04	0,64	64
**Hepatitis C**	Human Diseases	Infectious disease: Viral	59	164	0,35	10	0,57	0,62	1,30 E-04	0,64	65
**Cellular senescence**	Cellular Processes	Cell growth and death	65	182	0,35	10	0,57	0,61	7,12 E-05	0,64	66
**Gastric cancer**	Human Diseases	Cancers: Specific types	54	151	0,35	10	0,57	0,61	3,11 E-04	0,64	67
**Parathyroid hormone synthesis, secretion and action**	Organismal Systems	Endocrine system	41	111	0,36	10	0,57	0,63	8,76 E-04	0,64	68
**Toxoplasmosis**	Human Diseases	Infectious disease: Parasitic	41	112	0,36	10	0,57	0,63	1,07 E-03	0,64	69
**Lysine degradation**	Metabolism	Amino acid metabolism	30	63	0,47	9	0,43	1,10	3,40 E-05	0,63	70
**Breast cancer**	Human Diseases	Cancers: Specific types	52	150	0,34	10	0,57	0,59	8,76 E-04	0,63	71
**Measles**	Human Diseases	Infectious disease: Viral	51	147	0,34	10	0,57	0,59	9,62 E-04	0,63	72
**Protein processing in endoplasmic reticulum**	Genetic Information Processing	Folding, sorting and degradation	79	172	0,45	9	0,43	1,06	2,04 E-10	0,63	73
**Human papillomavirus infection**	Human Diseases	Infectious disease: Viral	115	361	0,31	10	0,57	0,54	3,02 E-05	0,63	74

**PI3K-Akt signaling pathway**	Environmental Information Processing	Signal transduction	114	364	0,30	10	0,57	0,53	7,40 E-05	0,62	75
**Th1 and Th2 cell differentiation**	Organismal Systems	Immune system	40	89	0,44	9	0,43	1,03	8,12 E-06	0,62	76
**Bladder cancer**	Human Diseases	Cancers: Specific types	24	41	0,58	8	0,29	2,03	4,10 E-06	0,62	77
**HIF-1 signaling pathway**	Environmental Information Processing	Signal transduction	51	116	0,43	9	0,43	1,01	1,16 E-06	0,62	78
**Th17 cell differentiation**	Organismal Systems	Immune system	46	105	0,43	9	0,43	1,01	4,10 E-06	0,62	79
**Herpes simplex virus**	Human Diseases	Infectious disease: Viral	70	219	0,31	10	0,57	0,54	1,22 E-03	0,62	80
**Galactose metabolism**	Metabolism	Carbohydrate metabolism	16	33	0,48	9	0,43	1,12	2,35 E-03	0,62	81
**Rap1 signaling pathway**	Environmental Information Processing	Signal transduction	69	217	0,31	10	0,57	0,54	1,49 E-03	0,62	82
**p53 signaling pathway**	Cellular Processes	Cell growth and death	31	72	0,42	9	0,43	0,99	2,44 E-04	0,62	83
**Adherens junction**	Cellular Processes	Cellular community - eukaryotes	39	71	0,54	8	0,29	1,90	3,06 E-08	0,61	84
**Metabolic pathways**	Metabolism	Global and overview maps	412	1591	0,25	10	0,57	0,44	7,52 E-05	0,61	85
**Fatty acid metabolism**	Metabolism	Lipid metabolism	26	62	0,41	9	0,43	0,96	1,22 E-03	0,60	86
**cAMP signaling pathway**	Environmental Information Processing	Signal transduction	68	219	0,30	10	0,57	0,53	3,10 E-03	0,60	87
**Cell cycle**	Cellular Processes	Cell growth and death	48	125	0,38	9	0,43	0,88	1,10 E-04	0,60	88
**C-type lectin receptor signaling pathway**	Organismal Systems	Immune system	39	114	0,33	10	0,57	0,58	5,20 E-03	0,60	89
**Longevity regulating pathway - multiple species**	Organismal Systems	Aging	26	64	0,40	9	0,43	0,93	1,99 E-03	0,59	90
**Melanoma**	Human Diseases	Cancers: Specific types	29	73	0,39	9	0,43	0,91	1,58 E-03	0,59	91
**ABC transporters**	Environmental Information Processing	Membrane transport	21	50	0,41	9	0,43	0,96	3,74 E-03	0,59	92
**Apoptosis**	Cellular Processes	Cell growth and death	45	137	0,32	10	0,57	0,56	6,04 E-03	0,59	93
**Cushing syndrome**	Human Diseases	Endocrine and metabolic disease	57	165	0,34	9	0,43	0,79	5,29 E-04	0,58	94
**Viral carcinogenesis**	Human Diseases	Cancers: Overview	76	226	0,33	9	0,43	0,76	1,44 E-04	0,58	95
**Lysosome**	Cellular Processes	Transport and catabolism	46	132	0,34	9	0,43	0,79	1,51 E-03	0,58	96
**Fluid shear stress and atherosclerosis**	Human Diseases	Cardiovascular disease	48	149	0,31	10	0,57	0,55	6,77 E-03	0,58	97
**N-Glycan biosynthesis**	Metabolism	Glycan biosynthesis and metabolism	24	52	0,45	8	0,29	1,59	4,16 E-04	0,58	98
**Central carbon metabolism in cancer**	Human Diseases	Cancers: Overview	27	70	0,38	9	0,43	0,88	3,74 E-03	0,57	99
**Leishmaniasis**	Human Diseases	Infectious disease: Parasitic	27	70	0,38	9	0,43	0,88	3,74 E-03	0,57	100
**GnRH signaling pathway**	Organismal Systems	Endocrine system	33	91	0,35	9	0,43	0,83	3,89 E-03	0,57	101
**Carbon metabolism**	Metabolism	Global and overview maps	42	123	0,33	9	0,43	0,78	3,74 E-03	0,56	102
**Amyotrophic lateral sclerosis**	Human Diseases	Neurodegenerative disease	103	374	0,27	11	0,71	0,37	1,38 E-02	0,56	103
**Apelin signaling pathway**	Organismal Systems	Circulatory system	44	137	0,31	10	0,57	0,55	1,02 E-02	0,55	104

**Ferroptosis**	Cellular Processes	Cell growth and death	17	40	0,42	9	0,43	0,97	8,58 E-03	0,55	105
**Hippo signaling pathway**	Environmental Information Processing	Signal transduction	49	160	0,30	11	0,71	0,42	1,62 E-02	0,55	106
**Cysteine and methionine metabolism**	Metabolism	Amino acid metabolism	21	53	0,39	9	0,43	0,91	8,31 E-03	0,54	107
**Thermogenesis**	Organismal Systems	Environmental adaptation	69	234	0,29	10	0,57	0,50	1,04 E-02	0,54	108
**Progesterone-mediated oocyte maturation**	Organismal Systems	Development and regeneration	32	94	0,33	10	0,57	0,58	1,27 E-02	0,54	109
**Valine, leucine and isoleucine degradation**	Metabolism	Amino acid metabolism	21	56	0,37	10	0,57	0,64	1,57 E-02	0,53	110
**Long-term potentiation**	Organismal Systems	Nervous system	25	70	0,35	10	0,57	0,61	1,57 E-02	0,52	111
**Amphetamine addiction**	Human Diseases	Substance dependence	26	71	0,36	9	0,43	0,84	9,90 E-03	0,52	112
**Cocaine addiction**	Human Diseases	Substance dependence	19	48	0,39	9	0,43	0,91	1,23 E-02	0,51	113
**Phosphatidylinositol signaling system**	Environmental Information Processing	Signal transduction	34	100	0,33	9	0,43	0,77	1,03 E-02	0,51	114
**Various types of N-glycan biosynthesis**	Metabolism	Glycan biosynthesis and metabolism	17	40	0,42	8	0,29	1,46	8,58 E-03	0,50	115
**cGMP-PKG signaling pathway**	Environmental Information Processing	Signal transduction	52	175	0,29	11	0,71	0,40	2,32 E-02	0,50	116
**Sphingolipid signaling pathway**	Environmental Information Processing	Signal transduction	40	129	0,30	11	0,71	0,42	2,48 E-02	0,49	117
**Cholesterol metabolism**	Metabolism	Lipid metabolism	19	49	0,38	9	0,43	0,89	1,55 E-02	0,49	118
**Terpenoid backbone biosynthesis**	Metabolism	Metabolism of terpenoids and polyketides	11	23	0,47	8	0,29	1,65	1,51 E-02	0,47	119
**Proximal tubule bicarbonate reclamation**	Organismal Systems	Excretory system	11	22	0,49	7	0,14	3,46	1,03 E-02	0,47	120
**IL-17 signaling pathway**	Organismal Systems	Immune system	31	94	0,32	10	0,57	0,56	2,27 E-02	0,46	121
**Biosynthesis of unsaturated fatty acids**	Metabolism	Lipid metabolism	14	34	0,40	9	0,43	0,94	2,32 E-02	0,44	122
**Propanoate metabolism**	Metabolism	Carbohydrate metabolism	14	34	0,40	9	0,43	0,94	2,32 E-02	0,44	123
**Wnt signaling pathway**	Environmental Information Processing	Signal transduction	48	164	0,28	12	0,86	0,33	3,75 E-02	0,44	124
**Sphingolipid metabolism**	Metabolism	Lipid metabolism	19	51	0,36	9	0,43	0,85	2,32 E-02	0,43	125
**RNA degradation**	Genetic Information Processing	Transcription	28	83	0,33	9	0,43	0,77	2,29 E-02	0,42	126
**Huntington disease**	Human Diseases	Neurodegenerative disease	84	308	0,26	11	0,71	0,37	3,30 E-02	0,42	127
**RNA transport**	Genetic Information Processing	Transcription	52	174	0,29	9	0,43	0,68	2,16 E-02	0,41	128
**Gap junction**	Cellular Processes	Cellular community - eukaryotes	29	87	0,32	9	0,43	0,76	2,32 E-02	0,41	129
**B cell receptor signaling pathway**	Organismal Systems	Immune system	26	78	0,32	10	0,57	0,57	3,25 E-02	0,39	130
**Glycerolipid metabolism**	Metabolism	Lipid metabolism	22	63	0,34	9	0,43	0,80	3,05 E-02	0,36	131
**Prion disease**	Human Diseases	Neurodegenerative disease	75	272	0,27	10	0,57	0,47	3,51 E-02	0,35	132
**Fatty acid degradation**	Metabolism	Lipid metabolism	19	53	0,35	9	0,43	0,82	3,49 E-02	0,34	133
**Fatty acid elongation**	Metabolism	Lipid metabolism	12	29	0,41	8	0,29	1,42	3,52 E-02	0,30	134

**Transcriptional misregulation in cancer**	Human Diseases	Cancers: Overview	60	214	0,27	10	0,57	0,47	4,51 E-02	0,28	135
**Hippo signaling pathway - multiple species**	Environmental Information Processing	Signal transduction	12	28	0,42	6	0,00	0,47	2,69 E-02	0,27	136
**Leukocyte transendothelial migration**	Organismal Systems	Immune system	36	118	0,30	8	0,29	1,04	4,24 E-02	0,21	137

Pathway analysis was performed using DIANA-miRPath v4.0 on all miRNAs identified as hnRNP A2b1-associated through motif search. **P-values** were adjusted using the Benjamini–Hochberg method (**FDR**). **Category** and **Subgroup** indicate the KEGG classification of each pathway. **Target genes** refers to the number of genes targeted by the miRNAs within a given pathway, while **Genes/Pathway (n***)* represents the total number of genes annotated in that pathway. **Norm. Coverage** was calculated by dividing the number of target genes by the total number of genes in the pathway, followed by min–max normalization. **miRNAs (n***)* indicates the number of miRNAs regulating the pathway, and **Norm. miRNAs** corresponds to their min–max normalized values. **Gene ratio** was calculated as normalized coverage divided by normalized miRNA number. **Score** integrates three normalized metrics—target gene coverage, miRNA count, and FDR significance. **Rank** indicates the relative significance of each pathway. The top 50 miRNAs illustrated in Figure 6 are highlighted in yellow in the table
