# Supplementary figures and images for: Ionizing Radiation Actively Reshapes Bone Marrow-Derived Extracellular Vesicle MicroRNA Cargo with the Involvement of hnRNP A2b1
Source: Int J Mol Sci. 2026 Jun 18;27(12):5510. doi: 10.3390/ijms27125510 (PMC13299799; doi:10.3390/ijms27125510)

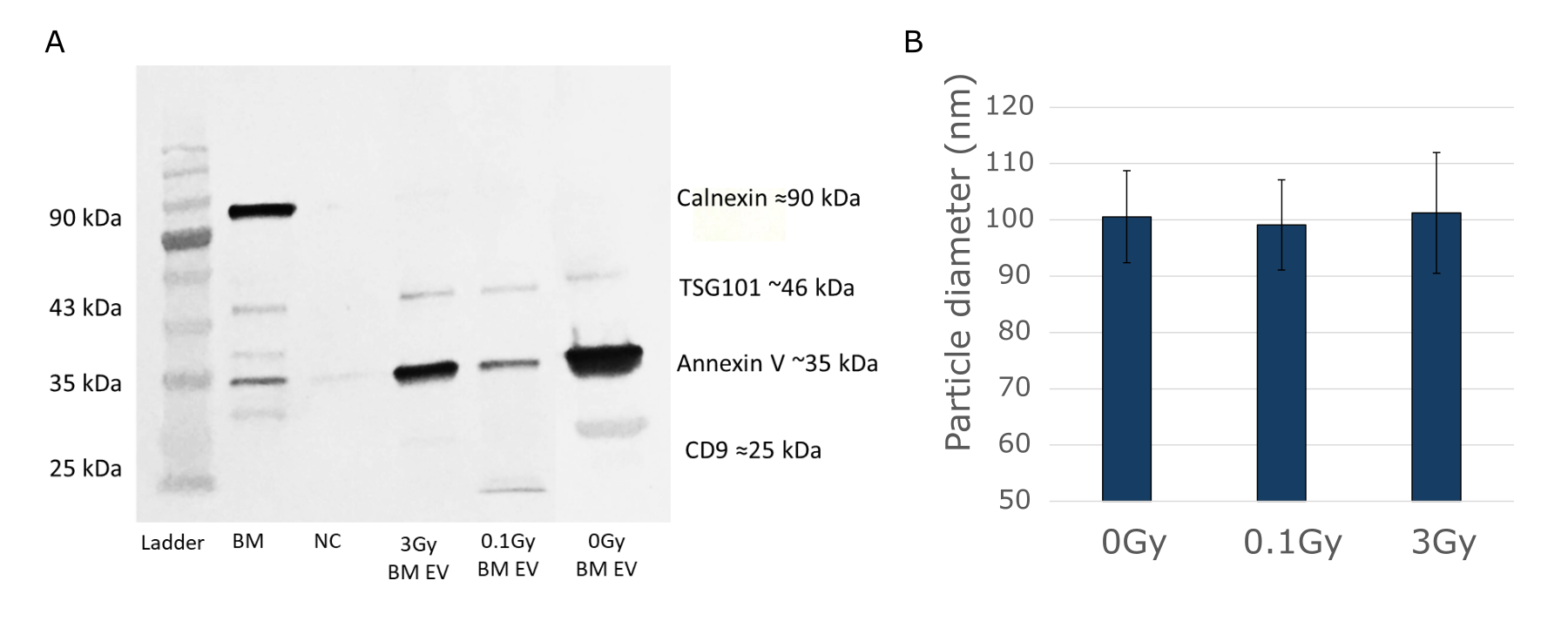

Supplement: Supplementary file 1 [file ijms-27-05510-s001.zip › Supplementary Figure1.jpeg]

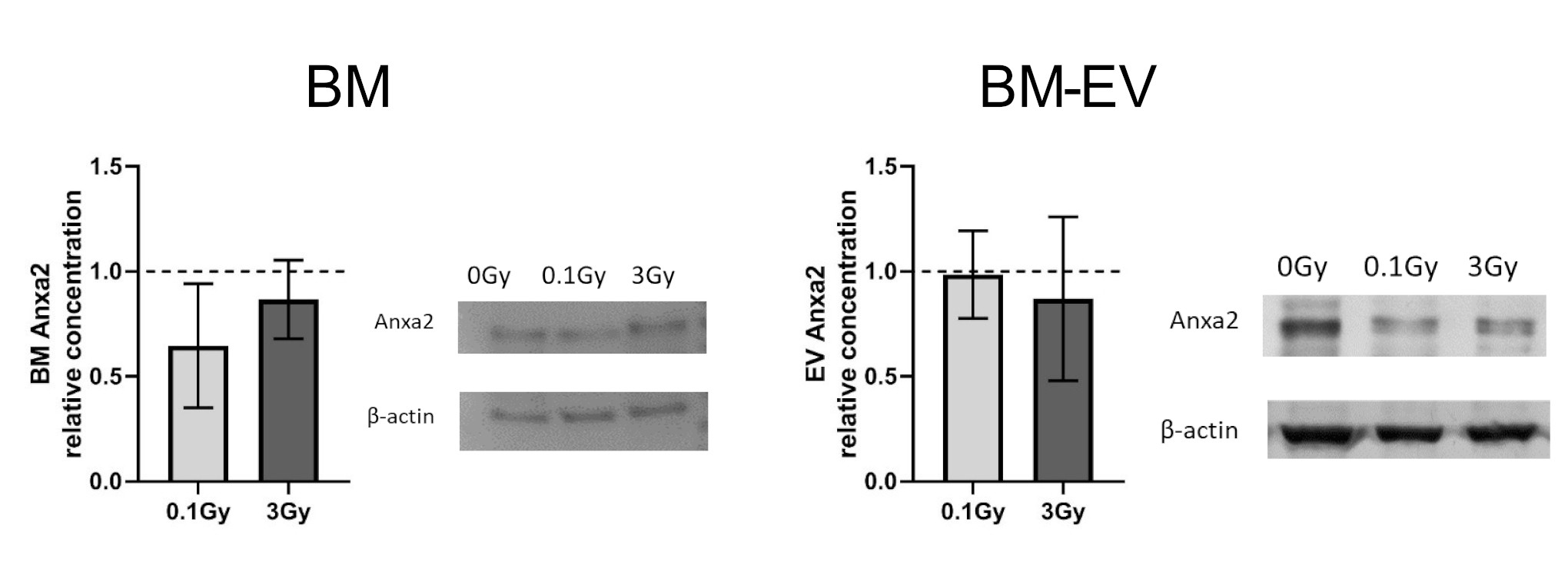

Supplement: Supplementary file 1 [file ijms-27-05510-s001.zip › Supplementary Figure2.jpeg]

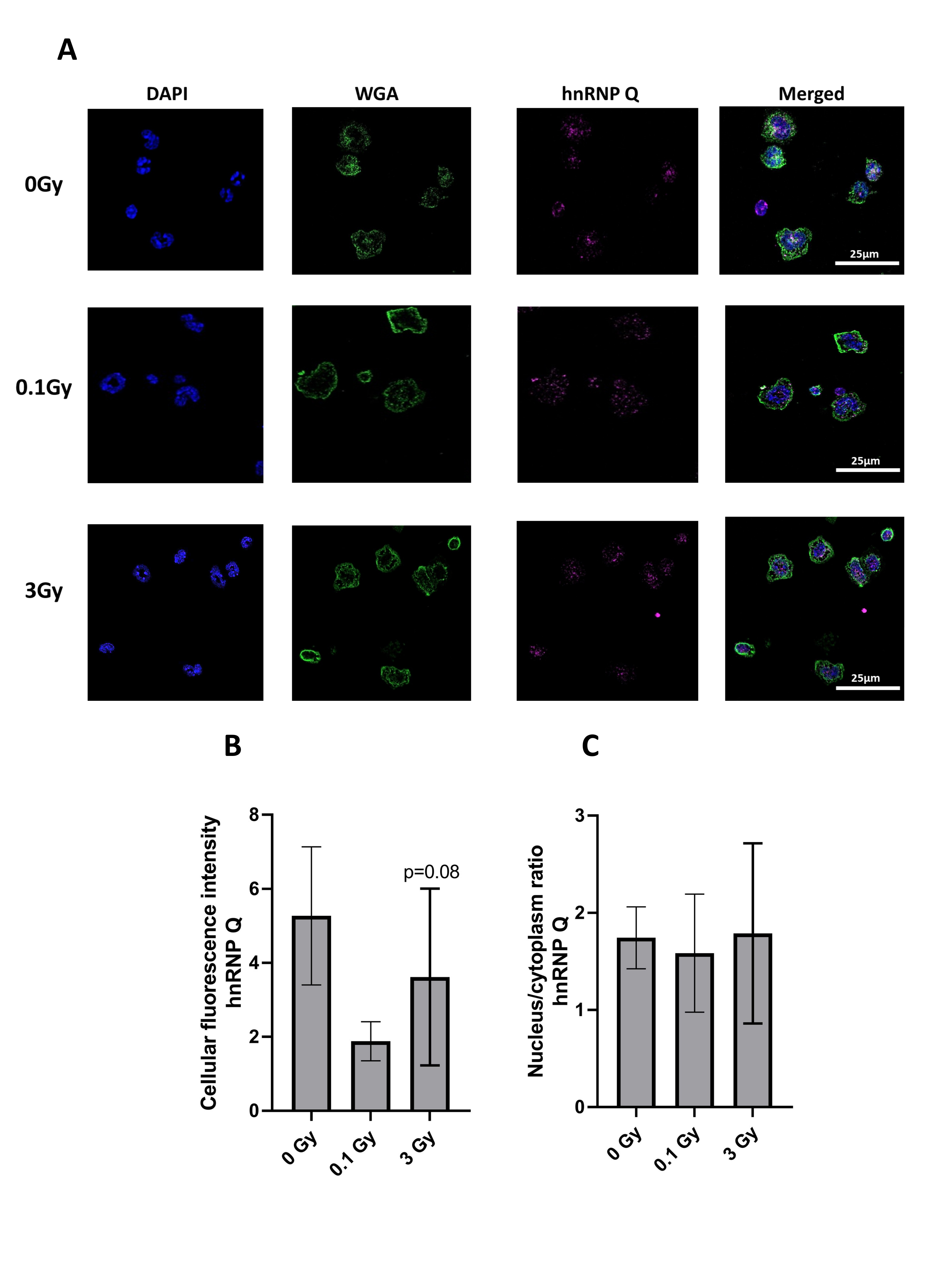

Supplement: Supplementary file 1 [file ijms-27-05510-s001.zip › Supplementary Figure3.jpeg]

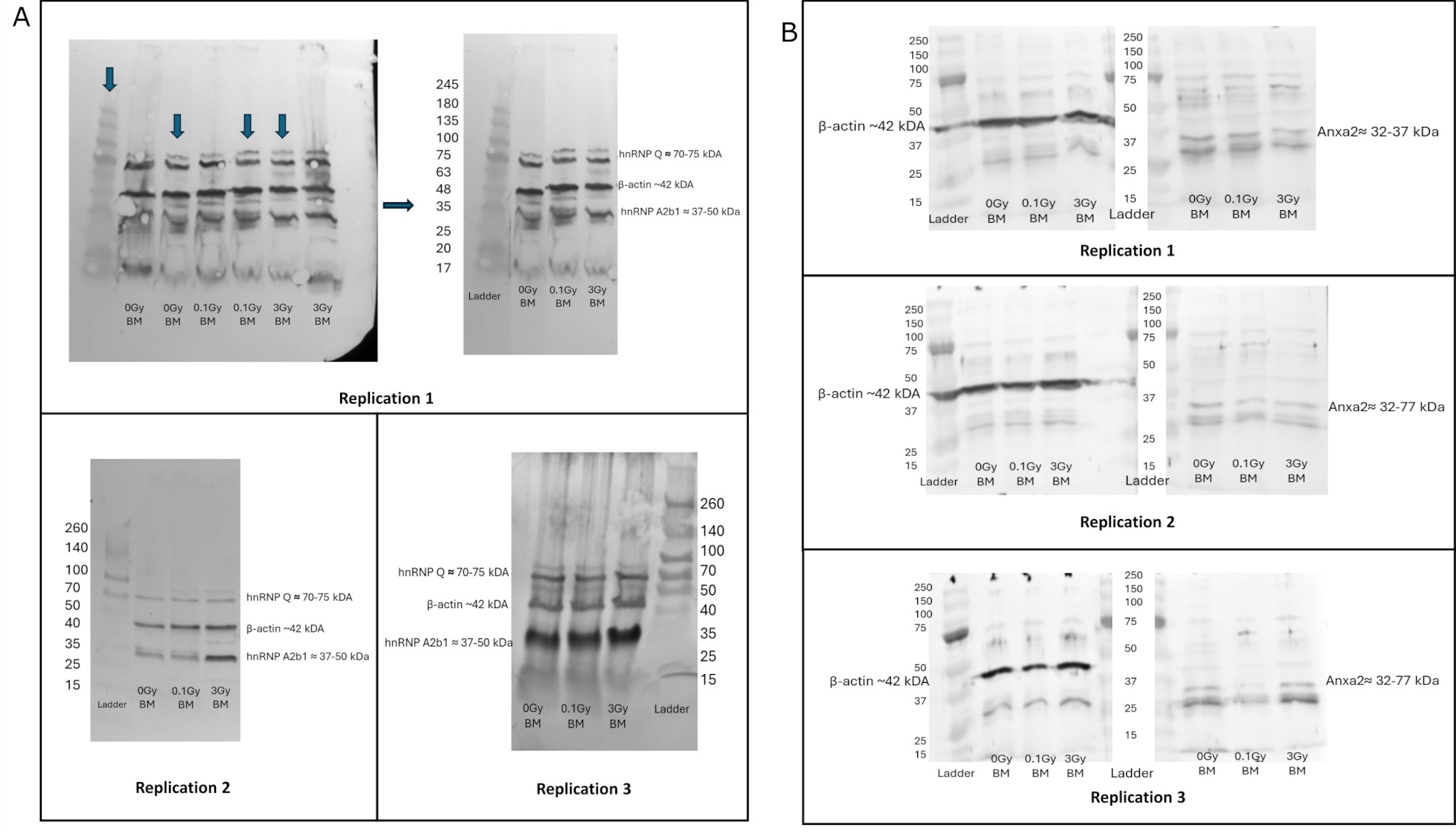

Supplement: Supplementary file 1 [file ijms-27-05510-s001.zip › Supplementary Figure4.jpeg]

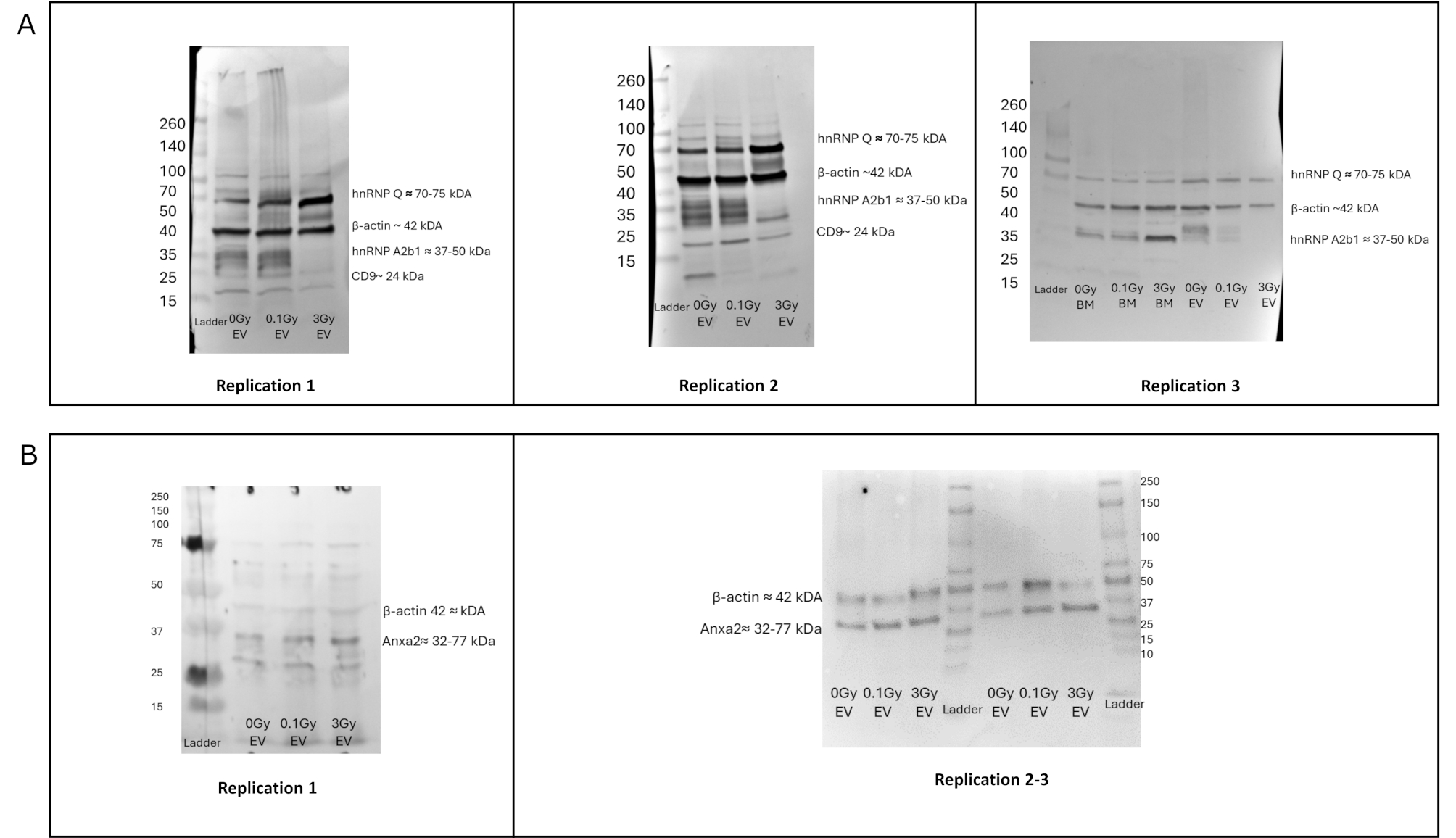

Supplement: Supplementary file 1 [file ijms-27-05510-s001.zip › Supplementary Figure5.jpeg]

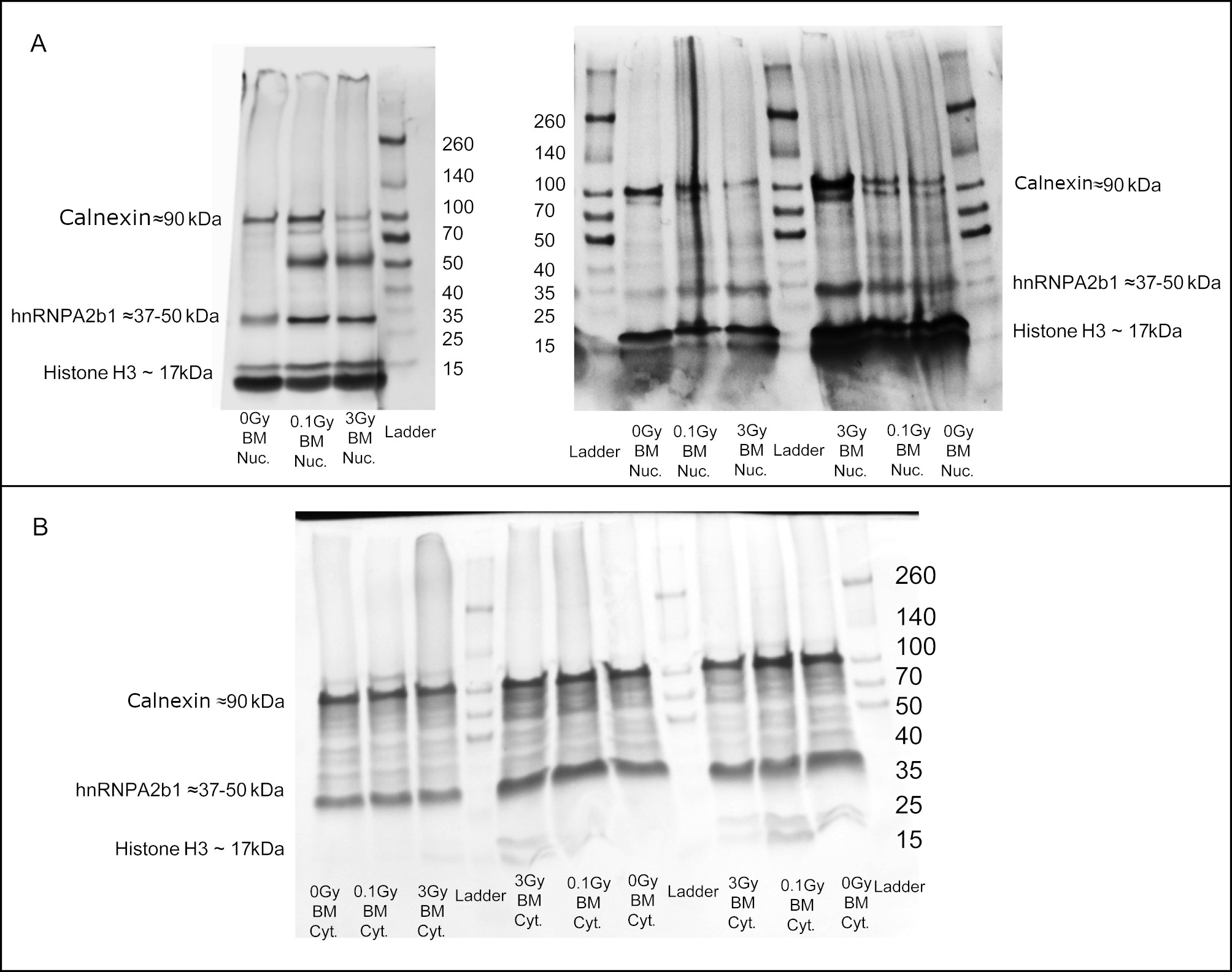

Supplement: Supplementary file 1 [file ijms-27-05510-s001.zip › Supplementary Figure6.jpeg]

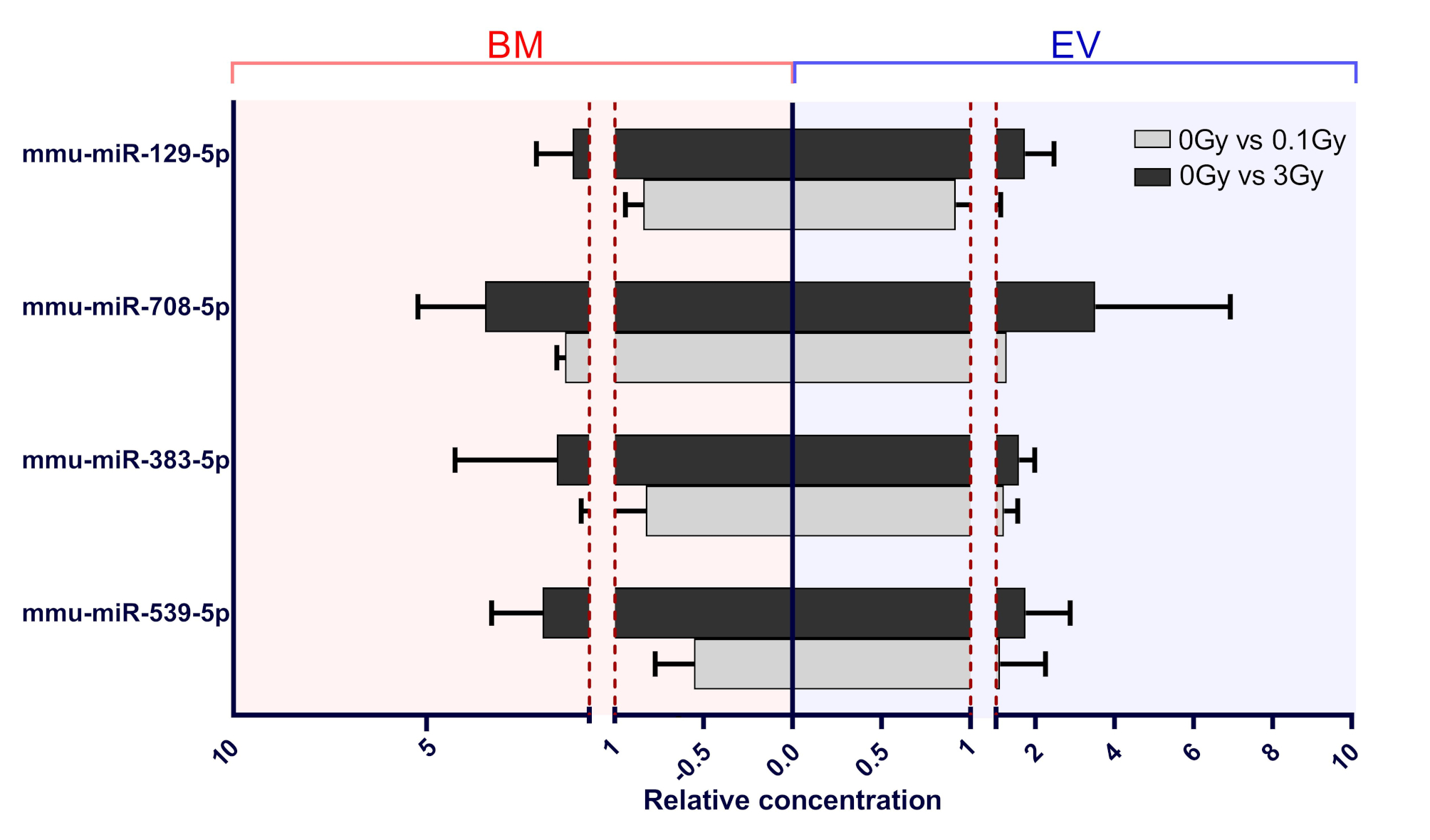

Supplement: Supplementary file 1 [file ijms-27-05510-s001.zip › Supplementary Figure7.jpeg]

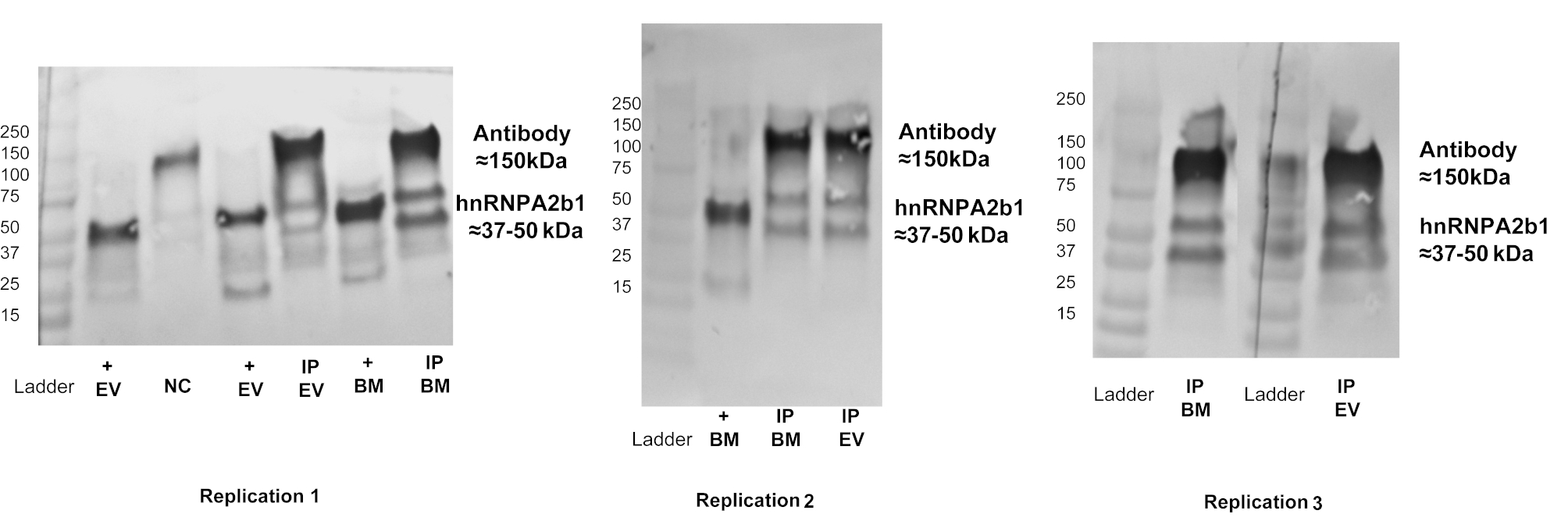

Supplement: Supplementary file 1 [file ijms-27-05510-s001.zip › Supplementary Figure8.jpeg]
